# Near infrared spectroscopy for cooking time classification of cassava genotypes

**DOI:** 10.3389/fpls.2024.1411772

**Published:** 2024-07-12

**Authors:** Massaine Bandeira e Sousa, Cinara Fernanda Garcia Morales, Edwige Gaby Nkouaya Mbanjo, Chiedozie Egesi, Eder Jorge de Oliveira

**Affiliations:** ^1^ Núcleo de Recursos Genéticos e Desenvolvimento de Variedades, Embrapa Mandioca e Fruticultura, Cruz das Almas, BA, Brazil; ^2^ Cassava Breeding Unit, International Institute of Tropical Agriculture (IITA), Ibadan, Oyo State, Nigeria; ^3^ Plant Breeding and Genetics Section, School of Integrative Plant Science, Cornell University, Ithaca, NY, United States; ^4^ National Root Crops Research Institute (NRCRI), Umuahia, Nigeria

**Keywords:** *Manihot esculenta* Crantz, classification models, portable NIR, accuracy, root quality

## Abstract

Cooking time is a crucial determinant of culinary quality of cassava roots and incorporating it into the early stages of breeding selection is vital for breeders. This study aimed to assess the potential of near-infrared spectroscopy (NIRS) in classifying cassava genotypes based on their cooking times. Five cooking times (15, 20, 25, 30, and 40 minutes) were assessed and 888 genotypes evaluated over three crop seasons (2019/2020, 2020/2021, and 2021/2022). Fifteen roots from five plants per plot, featuring diameters ranging from 4 to 7 cm, were randomly chosen for cooking analysis and spectral data collection. Two root samples (15 slices each) per genotype were collected, with the first set aside for spectral data collection, processed, and placed in two petri dishes, while the second set was utilized for cooking assessment. Cooking data were classified into binary and multiclass variables (CT4C and CT6C). Two NIRs devices, the portable QualitySpec® Trek (QST) and the benchtop NIRFlex N-500 were used to collect spectral data. Classification of genotypes was carried out using the K-nearest neighbor algorithm (KNN) and partial least squares (PLS) models. The spectral data were split into a training set (80%) and an external validation set (20%). For binary variables, the classification accuracy for cassava cooking time was notably high ( $R_{Cal}^{2}$ ranging from 0.72 to 0.99). Regarding multiclass variables, accuracy remained consistent across classes, models, and NIR instruments (~0.63). However, the KNN model demonstrated slightly superior accuracy in classifying all cooking time classes, except for the CT4C variable (QST) in the NoCook and 25 min classes. Despite the increased complexity associated with binary classification, it remained more efficient, offering higher classification accuracy for samples and facilitating the selection of the most relevant time or variables, such as cooking time ≤ 30 minutes. The accuracy of the optimal scenario for classifying samples with a cooking time of 30 minutes reached $R_{Cal}^{2}\;$ = 0.86 and $R_{Val}^{2}$ = 0.84, with a Kappa value of 0.53. Overall, the models exhibited a robust fit for all cooking times, showcasing the significant potential of NIRs as a high-throughput phenotyping tool for classifying cassava genotypes based on cooking time.

## 1 Introduction

With the increasing challenges associated with climate change, cassava (*Manihot esculenta* Crantz) has gained prominence as a crop of significant commercial relevance. Simultaneously, it maintains its high importance in the food security of millions of farmers, particularly in African countries (Ceballos et al., 2004). While all parts of the cassava plant are utilized for various purposes, cassava is primarily cultivated for its starchy roots, which can be used for human and animal feed, as well as in various industrial applications (Chiwona-Karltun et al., 2015). The roots are rich in carbohydrate (40% and 20% higher than rice and maize, respectively), providing consumers with an affordable source of calories (Bala et al., 2015).

The leaves and roots of cassava contain cyanogenic chemicals, which are the precursors to hydrocyanic acid (HCN), which in certain amounts can be poisonous or even fatal. Cultivars are categorized as sweet or table when they contain less than 100 mg kg^-1^ of cyanogenic compounds (wet basis), bitter cassava had a higher cyanogenic content (Wheatly et al., 2003). Sweet cassava is consumed fresh (cooked roots) or processed into various products such as cakes, snacks, and pies, etc. Boiling and frying cassava to serve as snacks is popular in Brazil. In Latin American and African countries, one of the simplest and most widespread methods to consume cassava roots is to boil them. For increased acceptance among end consumers, characteristics such as tenderness, mealy texture, or friability (ability to disintegrate between fingers and in the mouth) and short boiling time are essential (Hongbété et al., 2011; Bechoff et al., 2017).

Cooking time is a crucial determinant of the culinary quality of cassava roots. It holds high priority in cassava breeding programs due to its impact on energy and time savings during food preparation (Moreto and Neubert, 2014). Numerous environmental and genetic factors can influence cassava cooking time (Hongbété et al., 2011; Pedri et al., 2018; Iragaba et al., 2019; Silveira et al., 2021). A maximum acceptable time of thirty minutes has been established to optimize the commercialization and acceptance of cassava roots for culinary purposes in Brazil (Fukuda et al., 2002; Beleia et al., 2006; Vieira et al., 2018). Routine evaluation of root quality, including cooking time, is essential in the improvement of varieties targeted for the sweet cassava market (Iragaba et al., 2019).

Owing to their high starch content (20–35%), cassava roots culinary quality is greatly impacted by the physical and structural changes in starch that occur during cooking (Charoenkul et al., 2011). As starch undergoes gelatinization during cooking, absorbing water and leading to an increase in starch granule volume, these modifications result in a product with specific characteristics that are essential for consumer acceptance (Butarelo et al., 2004). In addition to starch structural properties, the maturity of the roots also impacts cooking time, which tends to be shorter in plants harvested at younger developmental stages (Santos et al., 2022). Therefore, these aforementioned factors should be taken into account by breeding programs when assessing cooking times.

Presently, the most widely method for assessing cassava cooking time is the softness test, which entail determining root tenderness using a fork after a specific time. An evolution in this process is the Mattson cooker, routinely used for determining the cooking time of peas, beans, and soybeans (Mattson, 1946; Jackson and Varriano-Marston, 1981). More recently, it has been adapted for cassava use (Miranda et al., 2008). This technique relies on metal rods with standardized weights exerting pressure as they pierce through cassava root pieces while they cook in boiling water. A more recent approach for assessing cassava cooking time is the water absorption method, which is faster, less subjective, and more reproducible than the softness test, which can take up to 60 minutes for some genotypes (Tran et al., 2021). The percentage of water absorption during boiling, calculated by the ratio of the initial root weight to the weight during boiling, correlates with cooking time and mealy texture, two crucial indicators of cooking quality (Beleia et al., 2004; Kouadio et al., 2011; Tran et al., 2021). All these methods are labor-intensive and time-consuming, taking over an hour to evaluate just one genotype. This contrasts with the realities of breeding programs that need to assess hundreds of samples daily in a quick and precise manner to proceed with the selection process. Thus, in order to improve the efficiency of cassava cooking assessment and analyze more samples in a shorter timeframe, it is imperative to develop and implement more effective methods.

The application of Near-Infrared Spectroscopy (NIRS) technologies has emerged as a game-changer in the term precision in phenotyping, revolutionizing selection stages within breeding programs. NIRS exhibits performance on par with conventional analytical chemistry methods, offering distinct advantages such as reduced analysis times, early assessment capabilities, the capacity to handle large sample volumes daily, and the non-destructive nature of sampling (Ikeogu et al., 2017). This sophisticated tool operates through the correlation of spectra information with reference data acquired through conventional methods to develop calibration models for predicting traits of interest (Pasquini, 2018; Saeys et al., 2019). The electromagnetic radiation in the NIRS region (700–2500 nm) is selectively absorbed by components like water and various organic compounds, including crucial elements such as carbohydrates, proteins, lipids, and alcohols (Agelet and Hurburgh, 2014). Consequently, NIRS stands out as a significant predictor of these compounds in organic substances.

NIRS spectroscopy has demonstrated remarkable efficacy in predicting cooking times across various species, including common beans (Cichy et al., 2015; Mendoza et al., 2018; Wafula et al., 2020, 2021) and rice (Thanathornvarakul et al., 2016). NIRS-based models have demonstrated strong predictive abilities, with validation R^2^ values greater than 0.87 and calibration R^2^ values greater than 0.81 (Thanathornvarakul et al., 2016). Critical properties of rice, including minimum cooking time, adhesiveness, pasting temperature, viscosity peak, and breakdown have been accurately predicted using NIRS-based model (Thanathornvarakul et al., 2016). In common beans, the NIRS-based prediction models for cooking time have exhibited exceptional precision, revealing an average prediction error of 8 minutes, underscoring the potential of NIRS in forecasting cooking times in bean varieties (Wafula et al., 2020). The NIRS technique has demonstrate efficacy in quantifying non-structural carbohydrates and was a suitable tool for analyzing the physiological responses of plants to diverse environmental stresses (Rosado et al., 2019).

Few cases of used of NIRS spectroscopy to predict cassava cooking have been recorded. A novel approach based on hyperspectral imaging for large-scale phenotyping has been recently developed (Meghar et al., 2023). This innovative approach meticulously process images for spectral data extraction and multivariate statistics are used to identified distinct regions of interest (Meghar et al., 2023). However, despite its potential, the results fail to accurately predict parameters linked to cooking ability. This limitation could stem from the relatively small sample size and the spectral data collection method, which involved fresh intact slices. Additionally, NIR hyperspectral imaging presents several drawbacks, including its high cost, the necessity for sensitive detectors for data collection, fast computers for analysis, and substantial data storage capacity requirements (Manley, 2014). Given this scenario, the present study proposes a new approach for classifying cassava genotypes based on cooking time through the development and validation of models using NIR spectroscopy. The result of this study will serve as a foundation for establishing a user-friendly protocol for NIR-based phenotyping of cooking time, which will facilitate the identification of genotypes that cook in predefined times that are more acceptable to end consumers.

## 2 Materials and methods

### 2.1 Plant material

A total of 888 cassava genotypes, which are part of the breeding program at Embrapa Mandioca e Fruticultura located in Cruz das Almas, Bahia, Brazil (12°39′25″ S, 39°07′27″W, 226 m altitude), were assessed. Seventeen trials (**Table 1**) were conducted during the crops seasons of 2019/2020, 2020/2021, and 2021/2022, in areas situated in the municipalities of Cruz das Almas, Laje (13°10’56”S, 39°25’30”W, 190 m altitude), Alagoinhas (12°7’13’’S, 38°24’35’’W, 151 m altitude), and Sátiro Dias (11°35’56’’S, 38°35’24’’W, 244 m altitude), all within the state of Bahia. The experiments were arranged using a complete randomized block design (with two or three replications) and an augmented block design (including 10 standard checks per block). The trials were conducted under rainfed conditions. Planting was performed using stakes that were 16–20 cm long and contained 5–7 buds each. Each plot consisted of two to six rows with 8–10 plants per row, spaced at 0.90 m between rows and 0.80 m between plants.

**Table 1:** Trials information with location and crop seasons where spectral data collection was conducted on cassava roots.

Trial name	Site	Year	Type of trial	Design	N° block/N° checks	Sampling NIR analysis*	
						# Genotypes	# Reads
BR.CETBAG.19.UFRB	Cruz das Almas, BA	2019/2020	CET	AB	9/10	424	2132
BR.PYTGS.19.PP1	Cruz das Almas, BA	2019/2020	PYT	RCB	2/6	110	688
BR.PYTGS.19NH2	Laje, BA	2019/2020	PYT	RCB	2/6	110	684
BR.PYTGS.19.RA1	Laje, BA	2019/2020	PYT	RCB	2/6	121	904
BR.CET.20.CNPMF	Cruz das Almas, BA	2020/2021	CET	AB	22/10	127	608
BR.CET.20.PP1	Cruz das Almas, BA	2020/2021	CET	AB	16/10	46	200
BR.AYTGS.20.NH1	Laje, BA	2020/2021	AYT	RCB	3/5	17	140
BR.AYTGS.20.PP1	Cruz das Almas, BA	2020/2021	AYT	RCB	3/5	30	364
BR.AYTGS.20.RA1	Laje, BA	2020/2021	AYT	RCB	3/5	30	356
BR.PTE.BAG.21.Candeal	Cruz das Almas, BA	2021/2022	PTE	AB	15/10	266	1343
BR.PYT.21.PP1	Cruz das Almas, BA	2021/2022	PYT	RCB	2/6	29	144
BR.PYT.21.SJ	Laje, BA	2021/2022	PYT	RCB	2/6	31	164
BR.PYTGS-C2.21.Emb	Cruz das Almas, BA	2021/2022	PYT	RCB	2/6	67	354
BR.PYTGS-C2.21.NH1	Laje, BA	2021/2022	PYT	RCB	2/6	41	188
BR.UYTGS.21.AlaBoaUniao	Alagoinhas, BA	2021/2022	UYT	RCB	3/5	9	108
BR.UYTGS.21.SDAP	Sátiro Dias, BA	2021/2022	UYT	RCB	3/5	11	124
BR.UYT.21.NH	Laje, BA	2021/2022	UYT	RCB	3/5	10	120

AB, Augmented block design; RCB, Randomized complete block design; PTE, Phenotypic evaluation trial; CET, Clonal evaluation trial; PYT, Preliminary yield trial; AYT, Advanced yield trial; UYT, Uniform yield trial; * Number of genotypes that were phenotyped for cooking time and collected spectral data.

All cultivation practices followed the guidelines outlined by Souza et al. (2006). The climate conditions in the regions are predominantly warm, humid, and tropical (Aw/Am, according to the Köppen classification), with an approximately 12-hour photoperiod throughout the year (Souza et al., 2020).

### 2.2 Sample preparation and cooking time analysis

The trials were harvested between the eleventh and the twelfth month after planting. Fifteen roots from three competitive plants per experimental plot (five healthy roots were selected from each harvested plant) with diameters ranging from 4 to 7 cm, were randomly selected for cooking analysis and spectral data collection. After classification, the roots were washed in running water and peeled. Two pieces from the central region of each root were collected. The first piece, which had a longitudinal length of around 10 cm with a variable diameter depending on the genotype, was used for the cooking analysis. The other portion, which was the same size, was put in Petri dishes after being cooked in a food processor and used to collect spectral data.

For the cooking time evaluation, five cooking times (CT): 15 (CT15m), 20 (CT20m), 25 (CT25m), 30 (CT30m), and 40 (CT40m) minutes after the onset of cooking were analyzed. Root samples were divided into three repetitions of five to nine pieces each and placed in a pot of boiling water (98°C), with a mass ratio of 1:10 (cassava: water). Cassava was considered cooked when little resistance to fork penetration perpendicular to their length was observed.

Cooking data were organized into binary and multiclass variables. For each cooking time, binary variables indicating cooked or uncooked were considered. Additionally, these same variables were used for multiclass classification based on 4 classes (CT4C) and 6 classes (CT6C) associated with cooking time. Classes that were taken into consideration for CT4C were, cooked at 20 min, 30 min, 40 min, or uncooked, while the following classes were considered for CT6C including cooked at 15 min, 20 min, 25 min, 30 min, 40 min, or uncooked.

To address the imbalance in class representation in binary variables, we employed the oversampling technique. This involved randomly duplicating readings from the underrepresented class (cooked). As a result, for spectral data classification analyses, an equal distribution of classes was ensured for each cooking time, with 50% of readings from roots that cook and 50% from roots that do not cook.

### 2.3 Data spectral collection and analysis

The spectral data were acquired from processed root samples housed in two Petri dishes. This procedure utilized a benchtop NIRFlex N-500 spectrometer (Büchi, Flawil, Switzerland) (NIR NIRFlex) and a portable device, QualitySpe® Trek, model S-10016 (NIRS QST). Every Petri dish was scanned twice, for a total of four repetitions for each experimental plot.

The NIRFlex N-500 spans a wavelength range of 1000–2500 nm (10000–4000 cm^-1^) and operates in diffuse reflectance mode, featuring a spectral resolution of 8 cm^-1^, interpolated at 4 cm^-1^, resulting in 1501 data points per spectrum. Employing a polarization interferometer with TeO_2_ wedges, a Tungsten Halogen lamp as the NIRS light source, and an InGaAs detector, this instrument delivers accurate spectral data. On the other hand, measurements was made using the portable QST device in diffuse reflectance over a wavelength range of 350–2500 nm, with a spectral resolution of 9.8 nm at 1400 nm. The QST device incorporates three detectors: 350–1000 nm (512-element silicon array), 1001–1785 nm (InGaAs photodiode), and 1786–2500 nm (InGaAs photodiode). This device is equipped with an internal light source and an internal gray scale reference for optimization and wavelength calibration, featuring a Quartz Tungsten Halogen bulb with a color temperature of 2870 K ±33 K. The window of the QST device, approximately 1 cm in diameter, is illuminated by the internal light source, and the internal design minimizes specular reflections.

Principal component analysis (PCA) was performed using the *prcomp* package (Kassambara and Mundt, 2020) on the centered and standardized (i.e., each wavelength was centered by subtracting the overall mean and standardized by dividing by the sample standard deviation) spectra data in order to assess the population structure of genotypes based on their individual cooking times. A symmetric biplot was generated using the Biplot function to graphically display the genotypes grouping.

The heritability of each wavelength from the spectral data was assessed for each trial. Variance components were estimated using a linear mixed model and the R package *lme4* (Bates et al., 2015). The model used for data analysis can be expressed as: $y=X_{b}+Z_{g}+e$ , where y represents the wavelength reflectance data, *b* represents fixed block effects, and *g* and *e* denote random effects of genotype and error, respectively. Matrices X and Z symbolize the incidence matrices for the respective b and g effects within the mixed model. All random effects were assumed to conform to a normal distribution, where $g∼N\left(0,\sigma_{g}^{2}\right)$ and $e∼N\left(0,\sigma_{e}^{2}\right)$ . Broad-sense heritability ( $H^{2}$ ) was then calculated using the formula: $H^{2}=\sigma_{g}^{2}/\left(\sigma_{g}^{2}+\sigma_{e}^{2}\right)$ . All statistical analyses were executed using R software version 4.2.3 (R Core Team, 2023).

### 2.4 Data pre-processing and classification model adjustment

Several pre-processing techniques were evaluated to ensure the reliability of spectral data, being used to enhance the signals of interest and at the same time remove or attenuate noise. The techniques including first-order derivative (1st), detrend (DT), multiplicative scatter correction (MSC), and combined methods such as first-order derivative-detrend (1st-DT), first-order derivative-multiplicative scatter correction (1st-MSC), detrend-multiplicative scatter correction (DT-MSC), and first-order derivative with Savitzky–Golay-detrend (1st-SG-DT). The first-order derivative was used to subtract the background and baseline drift, DT was employed to eliminate baseline drift in the spectra, and the MSC method was applied to eliminate multiplicative interference of scattering in the spectral signal.

Following pre-processing, the spectra were smoothed using an N=11 filter at each end of the spectral set for noise reduction (Savitzky and Golay, 1964). Data preprocessing was performed using *prospectr* package (Stevens and Ramirez-Lopez, 2022) implemented in R software version 4.2.3 (R Core Team, 2023).

After pre-processing, the spectral data were organized into a matrix X (predictors), and the cooking data were allocated in a vector Y (response). Two classification models were assessed for their potential in predicting cassava clones’ cooking times. The k-nearest neighbor algorithm (KNN) method (Mucherino et al., 2009) is based on determining distances (usually Euclidean) between an unknown object and each object in the training set. It is one of the most widely used non-parametric algorithms in machine learning due to its simplicity. The KNN method selects the smallest distance for assigning members of a particular class. The k-nearest objects (where k is the number of neighbors) of the unknown sample are chosen, and a majority rule is applied: the unknown sample is classified into the class to which the majority of the k objects belong. The choice of k is optimized by calculating the prediction capacity with different k values. The other method employed in the analyses, partial least squares (PLS), also known as projection to latent structures, models matrices X and Y simultaneously to find latent variables in X that will best predict latent variables in Y. These PLS components are similar to principal components. The models are implemented in the *caret* package (Kuhn, 2008).

### 2.5 Cross-validation and external validation

The data were split into a cross-validation set (80% of the data) and an external validation set (remaining data used to test classification models), both with an even distribution of genotypes between the two classes (cooked or uncooked) for binary variables and among the four or six cooking times (for multiclass variables).

The models’ performance was assessed through 10-repetition 5-fold cross-validation conducted on the training set. The overall effectiveness of binary classification models was evaluated based on specificity, sensitivity, and receiver-operating characteristic (ROC) analyses. Sensitivity measures the probability of the classifier achieving true positives $\left(\frac{tp}{tp+fn}\right)$ , while specificity measures the probability of achieving true negatives $\left(\frac{tn}{tn+fn}\right)$ , where *tp* corresponds to the number of correctly recognized examples of the class (true positives), tn the number of correctly recognized examples that do not belong to the class (true negatives); fp examples that were incorrectly assigned to the class (false positives), and fn examples not recognized as class examples (false negatives). ROC analyses were developed to assess the classification accuracy of a statistical model that classifies objects into two categories. An ROC curve is a graph of sensitivity on the y-axis against (1 - specificity) on the x-axis for various threshold values t. The diagonal 45° line connecting (0,0) to (1,1) is the ROC curve corresponding to random chance. The ROC curve for the gold standard is the line connecting (0,0) to (0,1) and (0,1) to (1,1). Generally, ROC curves fall between these two extremes. The area under the ROC curve is a summary measure that essentially gauges accuracy across the entire dataset.

For multiclass variables, the average values of accuracy, Cohen’s Kappa statistic (unweighted) (Cohen, 1960), specificity, sensitivity, and area under the ROC curve (AUC) obtained in each cross-validation repetition were considered. Accuracy was calculated as $\frac{tp+t}{tp+fn+fp+tn}$ , and the Kappa index is based on the number of concordant responses defined by $\frac{p_{o}+p_{e}}{1-p_{e}}$ where $p_{o}$ is the proportion of units that agreed, and $p_{e}\;$ is the proportion of units for which agreement is expected by chance.

The external validation sample set comprised 20% of the spectral data. Prediction performance was evaluated with parameters generated from a confusion matrix. The parameters for assessing model quality included accuracy, Kappa index, sensitivity, and specificity.

## 3 Results

### 3.1 Initial cooking analyses of field trial samples and spectral heritability

In this study, 36% (N=319) of the evaluated genotypes displayed the ability to be cooked within 30 minutes in at least one environment. Notably, 85 genotypes, comprising 65 traditional cultivars, 53 local varieties, and 26 newly developed genotypes from the breeding program, exhibited significant cooking potential within 15 minutes. Among these, five traditional sweet cultivars (BRS Jari, BRS Gema de Ovo, BRS Dourada, and BRS 396) distinguished themselves for their elevated beta-carotene content, a precursor of provitamin A, coupled with low hydrogen cyanide (HCN) levels. Moreover, there exists a cultivar initially designed for industrial purposes (BRS Kiriris) due to its notable fresh root and starch yield, yet it also finds consumption in various regions owing to its intermediate cyanogenic compound levels. Beyond these cultivars, 53 local varieties and 26 newly developed genotypes from the breeding program exhibited promising cooking potential within the 15-minute timeframe.

**Figure 1:**
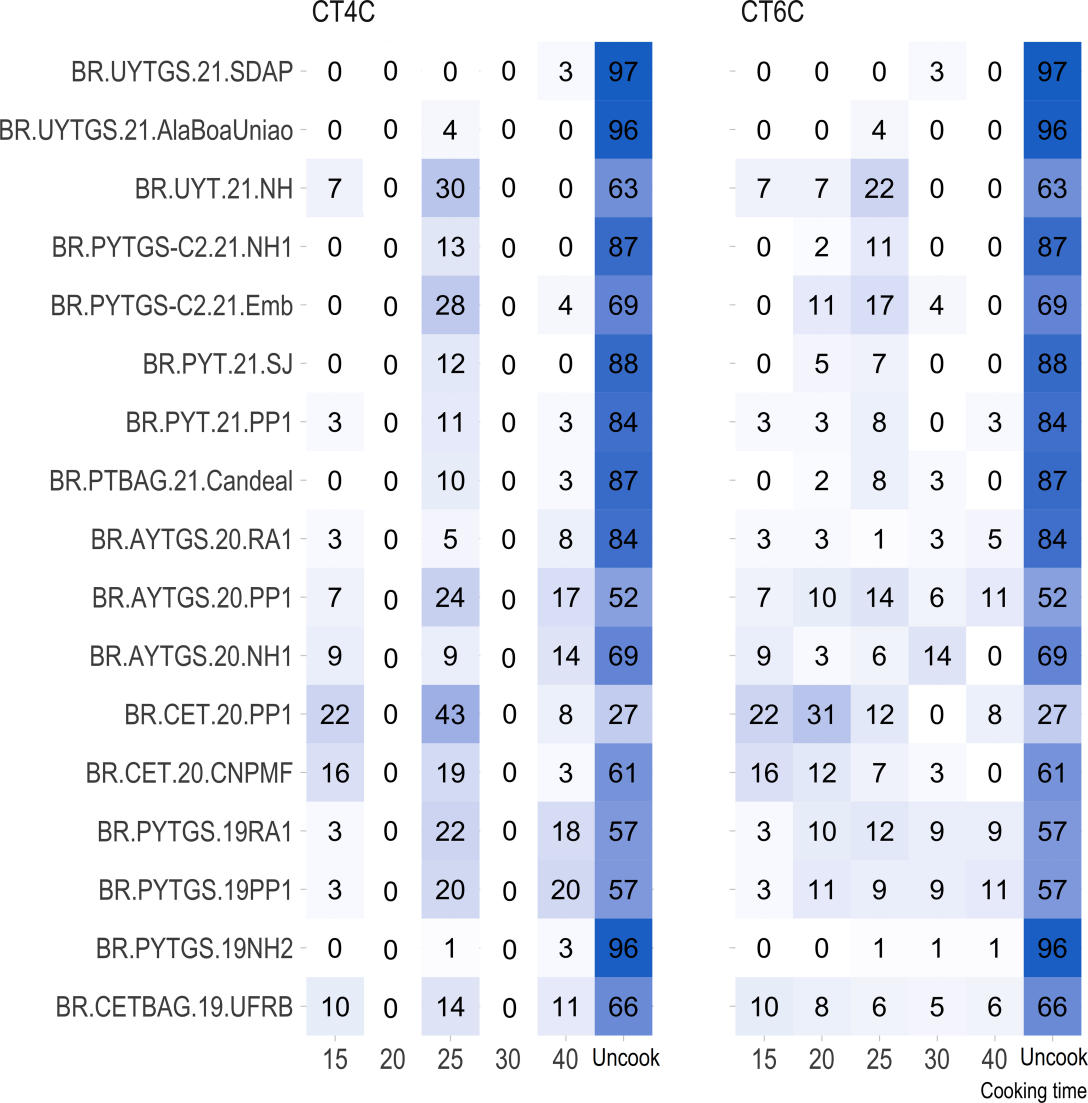
Percentage distribution of genotypes with roots cooked at various time intervals (15, 20, 25, 30, and 40 minutes) and genotypes with uncooked roots (NC) for each trial.

For most trials, the proportion of uncooked samples within 40 minutes was higher (> 50%) than those that cooked, except for the clonal trial BR.CET.20.PP1 (**Figure 1**).

**Figure 2:**
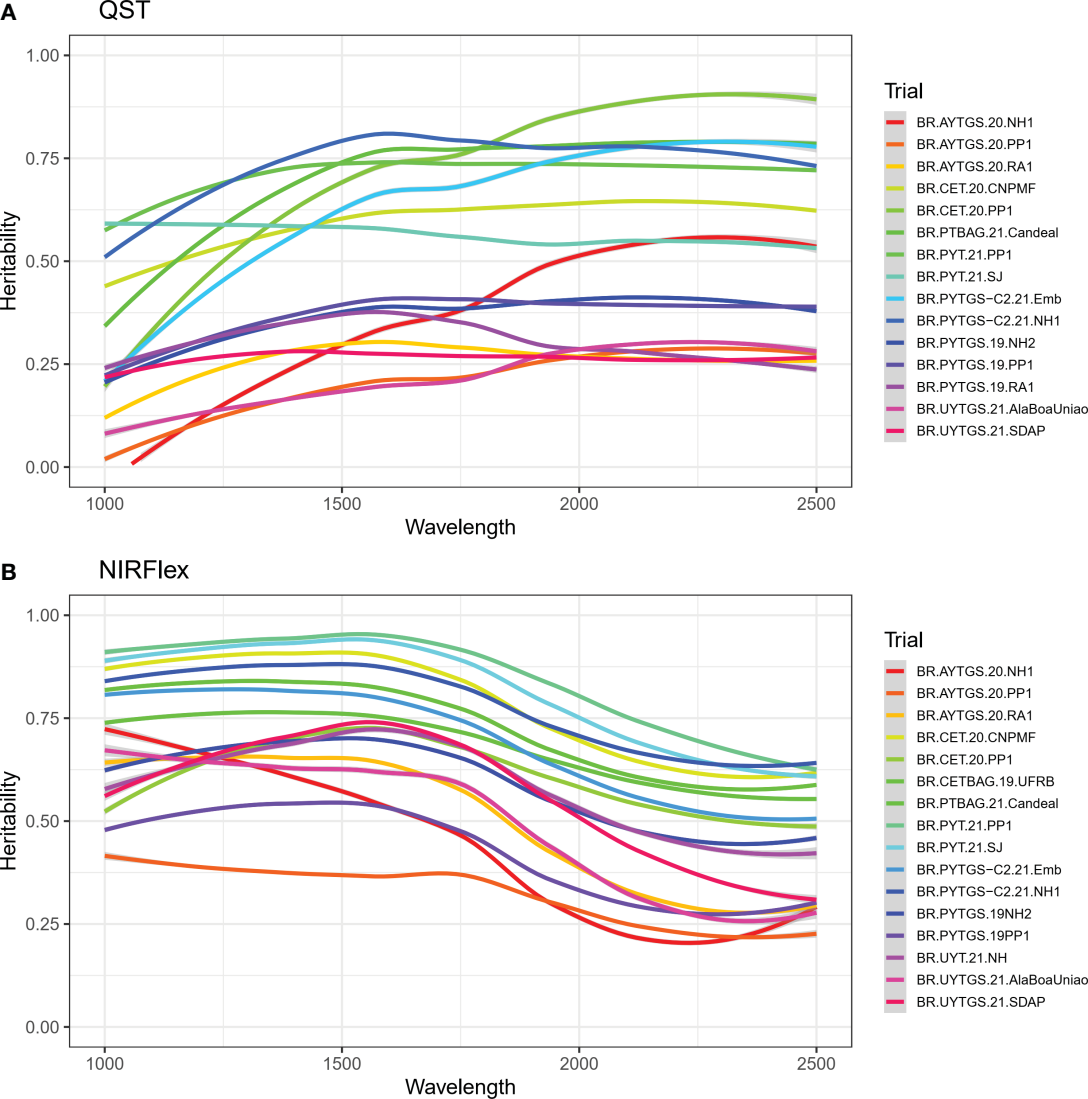
Broad-sense heritability for each wavelength of near-infrared spectra (NIRS) from cassava roots assessed for cooking ability using NIRS QualitySpec® Trek (QST) **(A)** and NIRFlex N-500 (NIRFlex) **(B)**.

The heritability of reflectance values was investigated for each trial. There were difference in the heritability of NIRS spectra across trials and spectral regions (**Figure 2**). Notably, trials BR.PYT.21.PP1 and BR.PYTGS-C2.21.NH1 which demonstrated higher heritabilities with values ≥ 0.70 for NIRS QST (**Supplementary Table S1**). For NIRFlex, trials BR.PYT.21.PP1 and BR.PYT.21.SJ displayed heritabilities exceeding 0.81. Trial BR.AYTGS.20.PP1 on the other hand had the lowest average heritabilities ( $H^{2}=18$ for NIRS QST, $H^{2}$ = 0.32 for NIRFlex) (**Supplementary Table S1**). Heritability was often significantly higher for NIRFlex, especially in the 1000 -1250 nm spectral range (**Figure 2**).

### 3.2 Principal component analysis of spectral data

A total of 2150 and 1500 wavelengths from NIRS QST and NIRFlex, respectively, were used in principal component analysis (PCA) (**Supplementary Figure S1**). There was variation among evaluated cassava genotypes, with minor differences between different cooking times (**Supplementary Figures S1**, **S2**). Overlapping classes were also observed for cooking times in different spectral ranges for the two NIR devices. The first principal component (PC) accounted for a variation of 79.65% (350 to 700 nm) to 98.60% (701 to 1000 nm) for NIRS QST (**Supplementary Figure S1**) and over 94.65% in both spectral ranges (1001–1750 nm and 1751–2500 nm) for NIRFlex (**Supplementary Figure S2**).

### 3.3 Efficiency of pre-processing methods and classification models

The accuracy, sensitivity, and specificity of the KNN classification method were used to assess the efficiency of pre-processing techniques (**Supplementary Figure S3**). When compared to using raw spectral data, the performance of the classification model was enhanced by the application of pre-processing techniques, as indicated by cross-validation results. The 1st + MSC methods were selected because they provided better accuracy, sensitivity, and specificity among binary and multi-class variables (**Supplementary Figure S3**).

**Figure 3:**
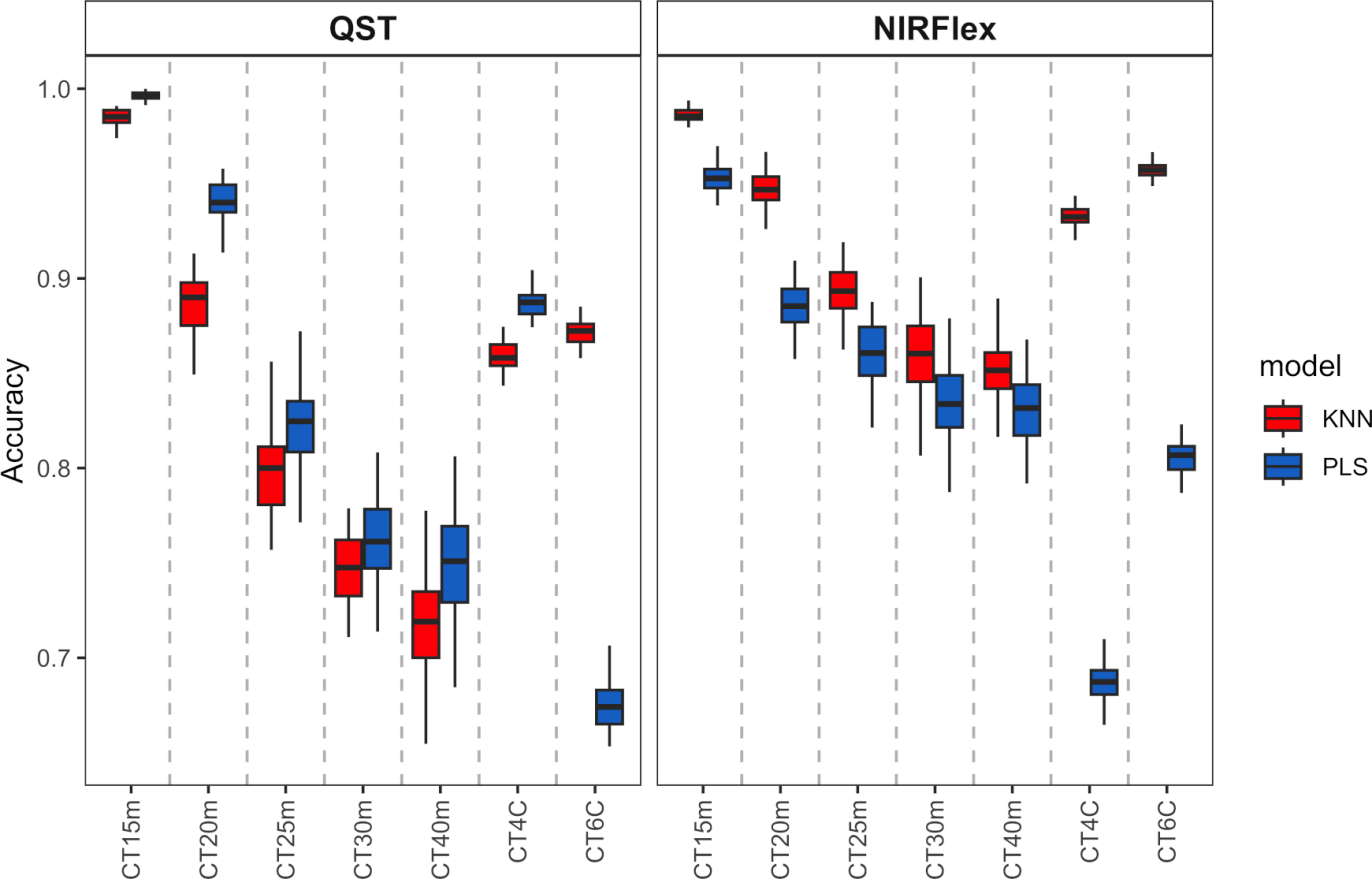
Cross-validation accuracy of cassava root classification models considering the cooking classification into binary variables (T15min, T20min, T25min, T30min, and T40min) and multiclass (CT4C and CT6C) based on near-infrared spectroscopy (NIR) spectra collected on NIRFlex N-500 (NIRFlex) and QualitySpec® Trek (QST) instruments.

Overall, the KNN and PLS models’ classification accuracy for binary variables was comparable. According to cross-validation, QST, accuracy ranged from 0.72 (KNN, T40min) to 1.00 (PLS, T15min) (**Figure 3**). The accuracy for NIRFlex were higher, ranging from 0.83 (PLS, T30min and T40min) to 0.99 (PLS, T15min).

Regarding the classification of cooking as multi-class variables, there was a more pronounced difference between classification models, where KNN outperformed PLS (variation from 0.96 to 0.87) except for CT4C with NIRS QST spectra, where both models were similar (variation from 0.67 to 0.81) (**Figure 3**).

With the exception of CT4C with QST spectra, for which both models performed similarly (variation from 0.67 to 0.81), there was a more pronounced difference between the classification models when it came to the classification of cooking as multi-class variables. KNN outperformed PLS in this regard (variation from 0.96 to 0.87) (**Figure 3**).

The results consistently showed stable probabilities of classifying samples into their respective categories across 50 repetitions of cross-validation (folds vs repetitions), as depicted in **Supplementary Figures S3** to **S10**. This stability held true for both binary and multiclass variables related to cooking time.

**Figure 4:**
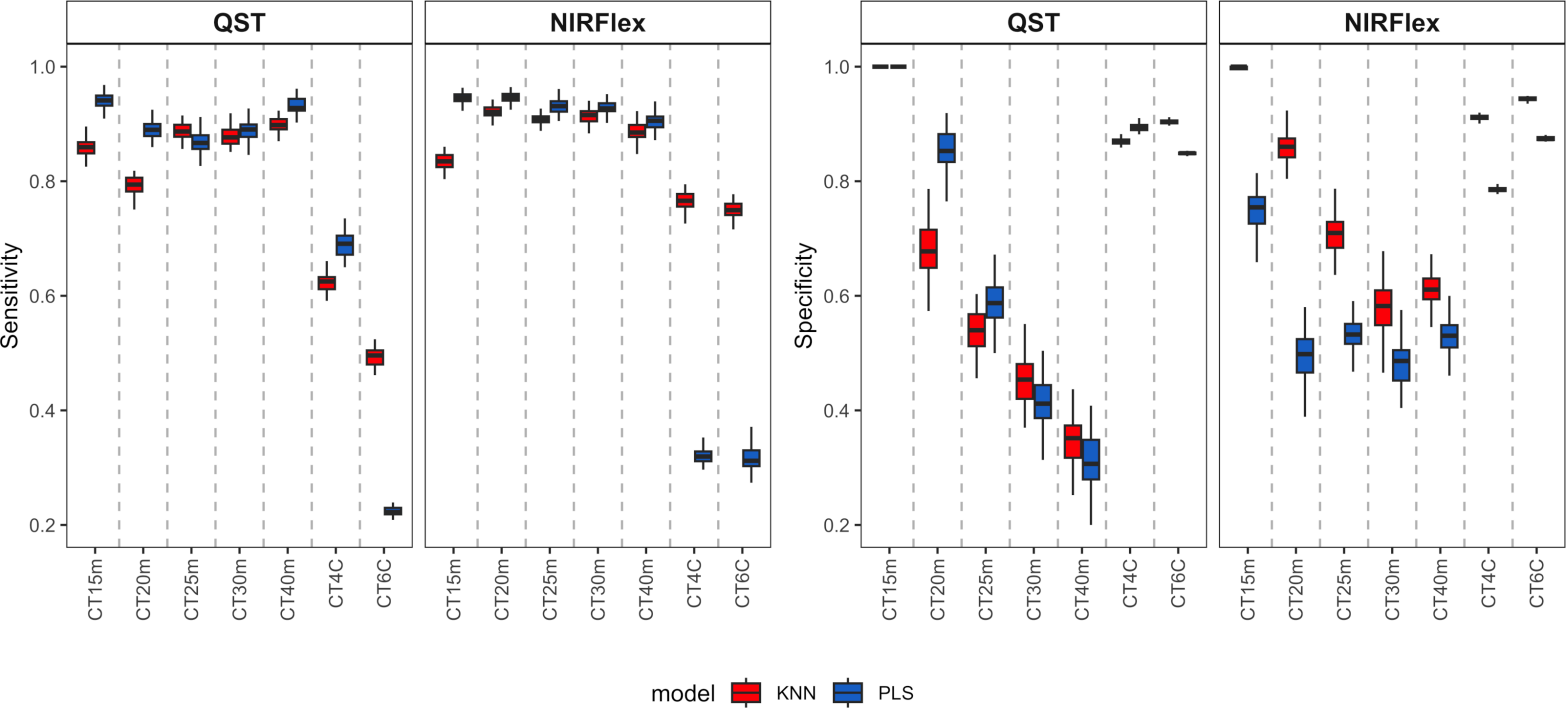
Sensitivity and specificity of cross-validation for cassava root classification models considering the cooking classification into binary variables (T15min, T20min, T25min, T30min, and T40min) and multiclass variables (CT4C and CT6C) based on near-infrared spectroscopy (NIR) spectra collected using NIRFlex N-500 (NIRFlex) and QualitySpec® Trek (QST) instruments.

Sensitivity displayed a pattern that was comparable to accuracy, with similarities between the PLS and KNN models for binary variables and significant differences for multi-class variables (**Figure 4**). For binary variables, sensitivity was approximately 0.90 for both instruments, indicating a high ability to classify the positive class (samples that are not cooked). For the multiclass variables CT4C and CT6C, uncooked samples were also considered as the positive class. Sensitivity values obtained by QST were 0.62 (KNN) and 0.69 (PLS) for CT4C and 0.49 (KNN) and 0.22 (PLS) on average for CT6C (**Figure 4**). For NIRFlex, the models showed comparable performance between the two multi-class variables, however the KNN model (~0.76) demonstrated a higher ability to classify the positive class compared to PLS (0.32) (**Figure 4**).

Specificity measures the ability of a model to predict true negatives for each available class. As this class (samples that are cooked) was underrepresented, the tendency is for specificity to be lower than sensitivity. Thus, specificity varied among the cooking times of binary variables, ranging from 0.31 (PLS, CT40m) to 1.00 (PLS and KNN, CT15m) (**Figure 4**). Therefore, the longer the cooking time, the lower the specificity values. For multiclass variables, specificity was close to 0.90 for QST and ranged from 0.80 to 0.95 for NIRFlex (**Figure 4**). Thus, for this type of variable, there was a greater balance in predicting true positives and negatives.

### 3.4 External validation of classification models

**Figure 5:**
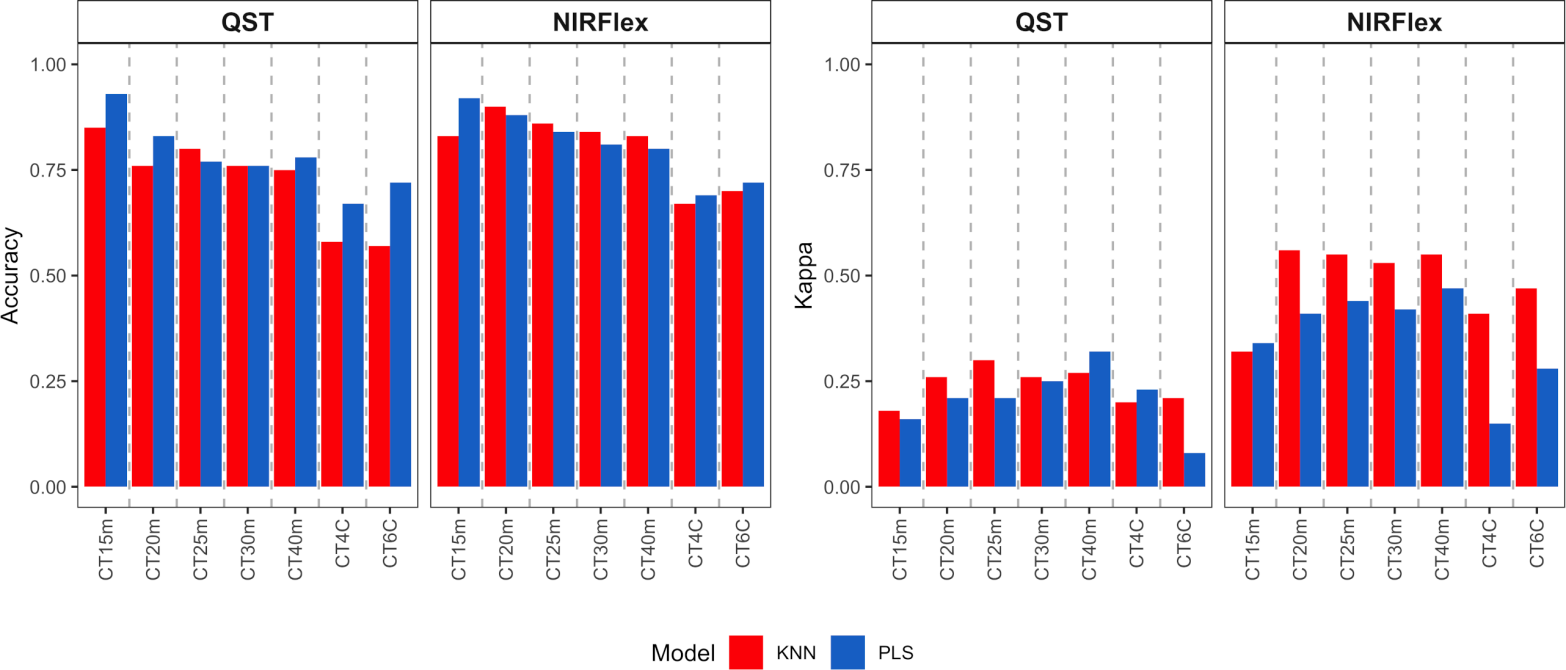
Accuracy of cassava root classification models in the external validation population, considering binary variables of cooking time (CT15m, CT20m, CT25m, CT30m, and CT40m) and multiclass variables (CT4C and CT6C) based on near-infrared spectroscopy (NIR) spectra collected using NIRFlex N-500 (NIRFlex) and QualitySpec® Trek (QST) instruments.

**Figure 6:**
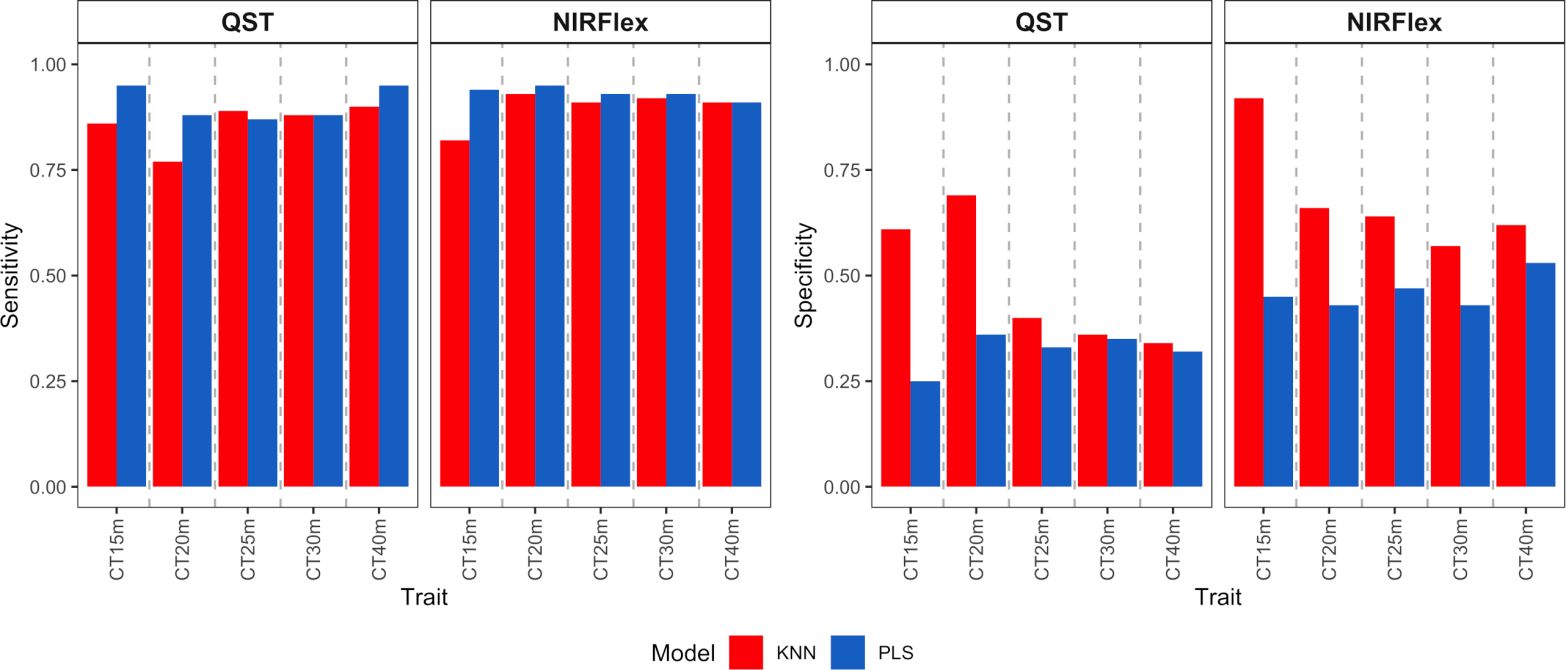
Sensitivity and specificity of cassava root classification models in the external validation population, considering binary variables of cooking time (CT15m, CT20m, CT25m, CT30m, and CT40m) based on near-infrared spectroscopy (NIR) spectra collected using NIRFlex N-500 (NIRFlex) and QualitySpec® Trek (QST) instruments.

**Figure 7:**
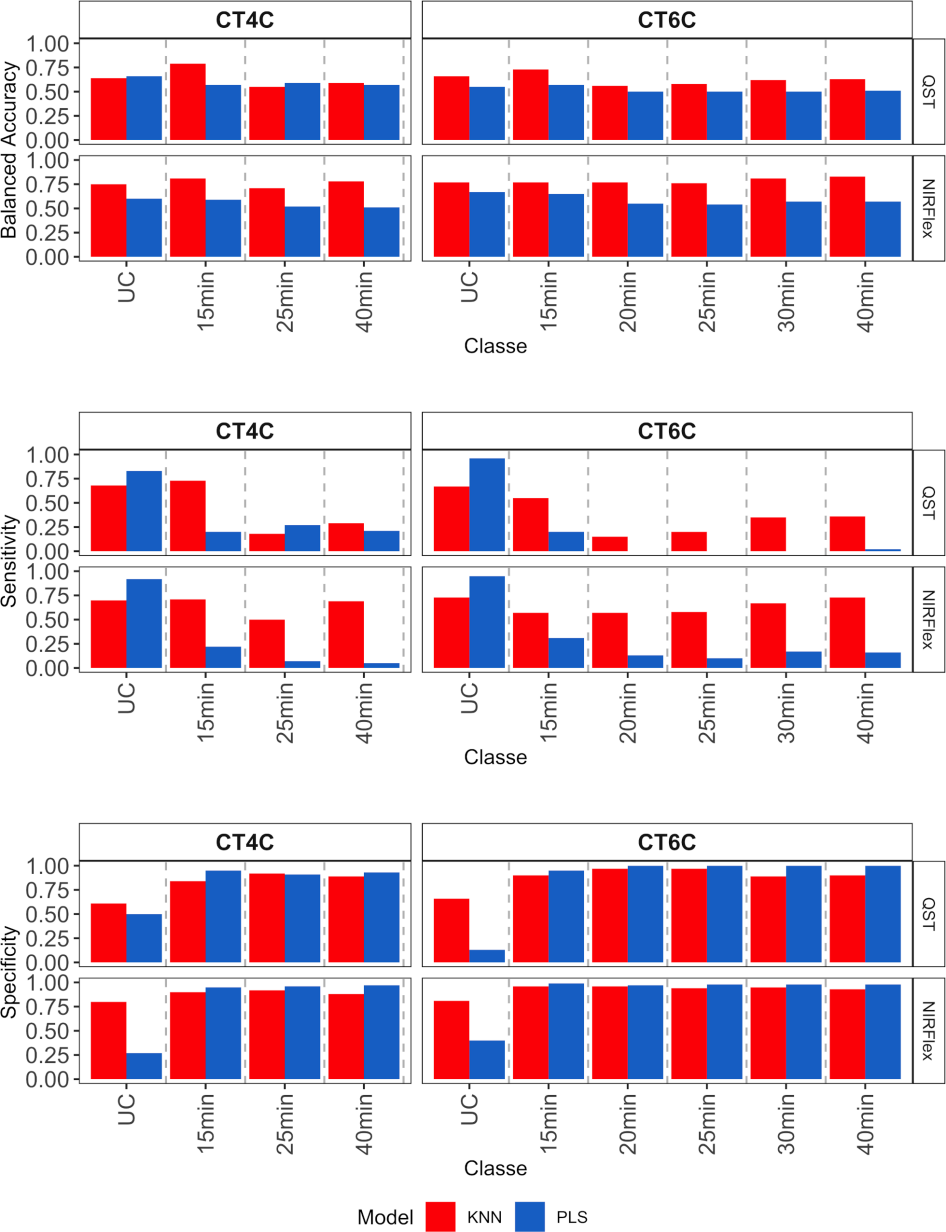
Parameters associated with the efficiency of cassava root classification models (accuracy, sensitivity, and specificity per class) in the external validation population, considering multiclass variables CT4C and CT6C based on near-infrared spectroscopy (NIR) spectra collected using NIRFlex N-500 (NIRFlex) and QualitySpec® Trek (QST) instruments.

External validation was conducted and assessed through the utilization of a confusion matrix, with a focus on quality parameters such as accuracy, Kappa index, sensitivity, and specificity across all variables (refer to **Figures 5**–**7**). The external validation population was formed by selecting 20% of the dataset. Both NIRS devices demonstrated a moderate to high magnitude accuracy, exceeding 0.57 for QST and 0.67 for NIRFlex (**Figure 5**). Looking more closely at the specifics, the PLS model consistently exhibited slightly higher accuracy values, ranging from 0.67 (QST, CT4C) to 0.93 (QST, CT15m) than the KNN model, which ranged from 0.57 (QST, CT6C) to 0.90 (NIRFlex, CT20m) (**Figure 5**). For Kappa index, it becomes evident that QST displayed inferior performance, with values ranging from 0.08 to 0.32, NIRFlex performance was more robust, with values spanning from 0.15 to 0.56. Overall, the KNN model consistently demonstrated higher Kappa values when compared with the PLS model.

For the binary variables, sensitivity was high for both instruments, with values ranging between 0.76 and 0.95 (**Figure 6**), while specificity values were lower, especially for the PLS model. Specificity varied from 0.25 to 0.69 (QST) and from 0.43 to 0.92 (NIRFlex) (**Figure 6**).

For multiclass variables, accuracy (balanced), sensitivity, and specificity parameters were obtained for each individual cooking time category (**Figure 7**). Overall, accuracy was similar across classes, models, and instruments (~0.63), although the KNN model showed slightly higher accuracy in the classification of all cooking time classes, except for the CT4C variable (QST), in the NoCook and 25 min classes (**Figure 7**). On the other hand, sensitivity varied among classes, with the KNN model performing better in the classification of multi-class variables (> 0.50, NIRFlex, and > 0.20 QST). For the PLS model, values close to zero were found for cooking times from 20 to 40 min for the CT6C variable (QST). This result indicates a lack of correct classification of true positives (samples that are cooking). For both instruments, specificity was higher than sensitivity (> 0.75), except for the category of samples that did not cook (**Figure 7**).

**Figure 8:**
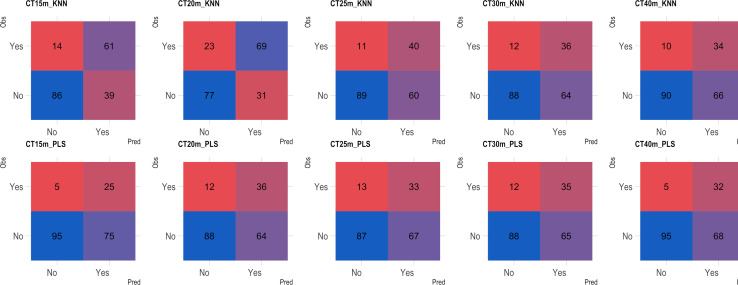
Confusion matrix of the external validation set considering classification models based on near-infrared spectroscopy by the QualitySpec® Trek (QST) instrument, evaluated on cassava roots considering binary variables of cooking time CT15m, CT20m, CT25m, CT30m, and CT40m. KNN, k-nearest neighbor algorithm; PLS, partial least squares.

**Figure 9:**
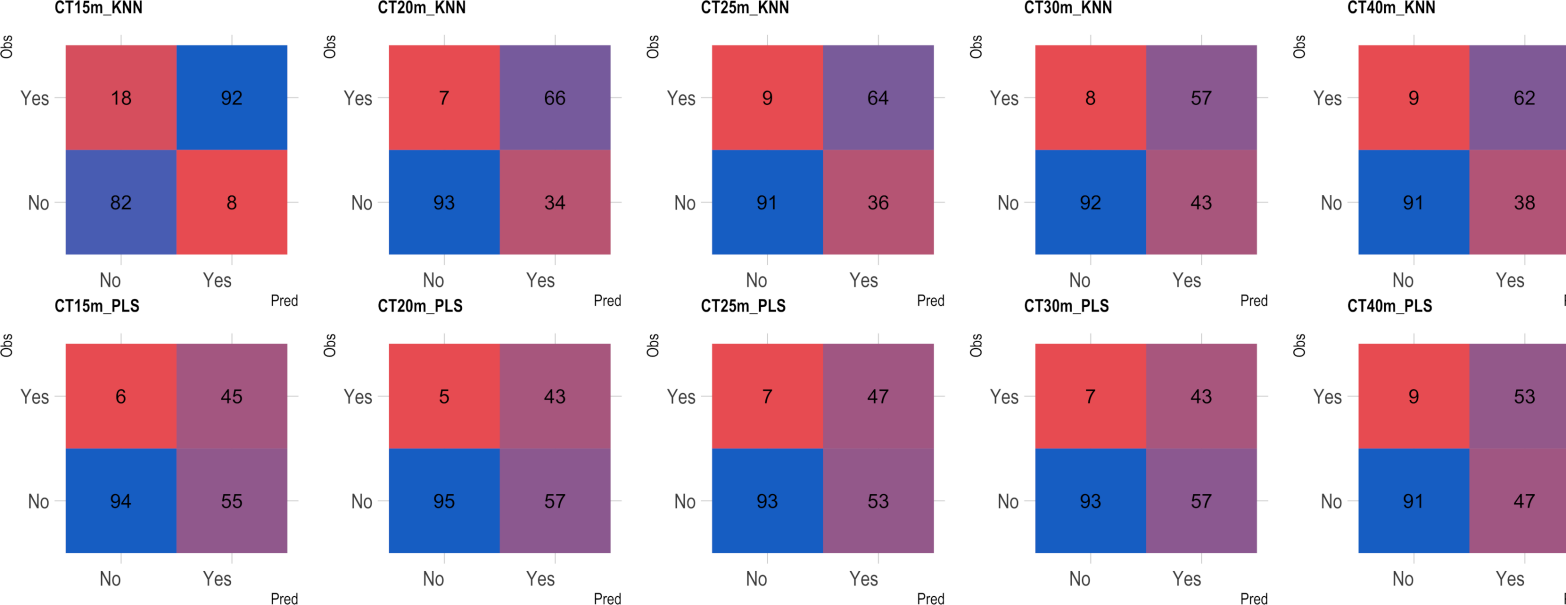
Confusion matrix of the external validation set, showcasing classification models based on near-infrared spectroscopy using the NIRFlex N-500 (NIRFlex) instrument, evaluated for cassava roots with binary variables of cooking time (CT15m, CT20m, CT25m, CT30m, and CT40m). KNN, k-nearest neighbor algorithm; PLS, partial least squares.

**Figure 10:**
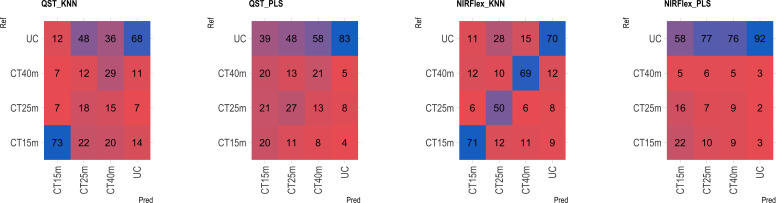
Confusion matrix of the external validation set, illustrating classification models based on near-infrared spectroscopy by the NIRFlex N-500 (NIRFlex) and QualitySpec® Trek (QST) instruments, evaluated for cassava roots considering multiclass variables of cooking time with 4 classes (CT4C). KNN, k-nearest neighbor algorithm; PLS, partial least squares.

**Figure 11:**
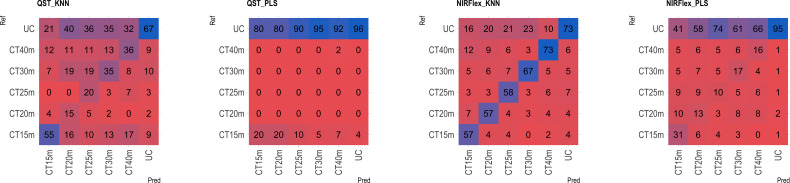
Confusion matrix of the external validation set, illustrating classification models based on near-infrared spectroscopy by the NIRFlex N-500 (NIRFlex) and QualitySpec® Trek (QST) instruments, evaluated for cassava roots considering multiclass variables of cooking time with 6 classes (CT6C). KNN, k-nearest neighbor algorithm; PLS, partial least squares.

Based on the analysis of the confusion matrix, the outcomes of classification by various models in the external validation set revealed a notable efficiency in categorizing clones that were not cooked, with accuracy percentages ranging from 77 to 95% (**Figures 8**–**11**) for both NIRS devices. However, divergences surfaced when it came to predicting which class of clones actually underwent cooking. In the case of QST, the KNN model displayed the highest accuracy percentages, ranging from 34% (CT40m) to 69% (CT20m), while the PLS model exhibited a narrower range, ranging from 25% (CT15m) to 36% (CT20m) (**Figure 8**). The accuracy percentages for NIRFlex were comparatively higher, ranging from 57% (CT30m) to 92% (CT15m) for the KNN model. The PLS model showed comparable values, hovering around 46% for all binary variables (**Figure 9**). Overall, the KNN model consistently outperformed the PLS model for the binary variables.

The multiclass variables CT4C and CT6C have comparable accuracy for identifying clones that do not undergo cooking for both NIR instruments. However, notable distinctions emerged among the models (**Figures 11**, **9**). The PLS model showed greater accuracy, ranging from 83% (CT4C, QST) to 96% (CT6C, QST) compared to the KNN model, which exhibited a range between 67% (CT6C, QST) and 73% (CT6C, NIRFlex) for clones that do not undergo cooking (**Figures 10**, **11**).

For clones cooked within 15 to 40-minute range, the KNN model (accuracy ranging from 15 to 73% in QST and 50 to 73% in NIRFlex) outperforming the PLS model (accuracy ranging from 0 to 27% in QST and 5 to 31% in NIRFlex) regardless of the number of multiclasses (**Figures 10**, **11**). When evaluating the performance of NIR instruments for the KNN model, the overall accuracy for classifying clones cooking up to 15 minutes was similar, with substantial differences between variables. The accuracy was higher when the data was divided into four classes (CT4C, 71%) compared to six classes (CT6C, 57%). The CT4C and CT6C classifications perform similarly for the KNN model for cooking times ranging from 20 to 40 minutes, with a noticeable differences between instruments. NIRFlex demonstrated higher accuracy ranging from 50% (25 minutes) to 73% (40 minutes), compared to QST, which ranged from 15% (20 minutes) to 36% (40 minutes). Overall, the most effective classification scenario was observed using NIRFlex, especially when the KNN model was applied on variables with fewer classes, like CT4C.

## 4 Discussion

### 5.1 Cooking time as a key characteristic for the selection of new sweet cultivars

In many countries in Latin America and the Caribbean, the most common form of consuming cassava is in the boiled root form. Characteristics such as root cooking time, associated with culinary quality and low cyanogenic potential (HCN), are crucial for end-user consumer acceptance (Bechoff et al., 2017; Ceballos et al., 2017) and attributes such as short cooking time become a priority over other agronomic and even nutritional characteristics. In recent years, cassava biofortification programs have prioritized boosting nutrient content, relegating cooking quality as a secondary trait (Ceballos et al., 2017). As a result, only a few clones with high carotenoid content, low HCN levels, and acceptable cooking quality have been obtained. This underscores the ongoing need for sustained efforts in crossbreeding to increase the number of segregating materials that align with consumer preferences in Latin America and the Caribbean (Ceballos et al., 2017).

Cooking time is difficult to measure. Conventional methods such as simple fork test or the use of penetrometers to measure root texture are time-consuming (around 60 minutes per sample) and labor-intensive, limiting the number of samples evaluated daily. Additionally, direct measurement of cooking time or texture requires a well-trained team with standardized protocols. Cassava breeding programs are actively exploring more efficient methods for assessing the cooking quality of cassava roots (Tran et al., 2021). One such method is the percentage of water absorption (WAB) during boiling, calculated by the ratio of initial root weight to the weight after boiling. WAB correlates indirectly with cooking ability, where a higher WAB percentage associates with shorter cooking times (Beleia et al., 2004; Kouadio et al., 2011; Tran et al., 2021). Using this method, Tran et al. (2021) evaluated 36 cassava genotypes and proposed two promising protocols for predicting cooking time based on water absorption and changes in relative density. These protocols are simple, objective, and up to 100 samples can be processed daily per operator. However, further tests are necessary to compare these methods and confirm their reliability across a broader range of genotypes.

NIRS is gaining popularity as a tool for evaluating food quality. In potatoes, image analysis was successfully in differentiating between cooked and uncooked parts, and spectral data has enabled development of a model for predicting ideal cooking times with less than 10% relative error (Trong et al., 2011). This methodology was recently applied to predict the water absorption percentage (WAB) and texture (area) of fresh root samples in cassava. However, the obtained R^2^ values (coefficient of determination) were low, ranging from 0.51 to 0.53, respectively (Meghar et al., 2023). Therefore, a standardized model based on spectral and hyperspectral imaging (HSI) for predicting WAB and, consequently, cooking time in cassava data is yet to be established.

Regardless of the methodology used, the maximum cooking time for cassava roots needs to be no more than 30 minutes to enhance the likelihood of new sweet cultivars being adopted in Brazil (Fukuda et al., 2002). In this study, 36% (N=319) of the evaluated genotypes could be cooked in at least one environment within 30 minutes. However, 85 genotypes (six five traditional cultivars, 53 local varieties, and 26 new genotypes from the breeding program) exhibited notable cooking potential within 15 minutes. Indeed, previous studies had already demonstrated the short cooking time of the cultivars BRS Gema de Ovo (13 minutes) and BRS Dourada (15 minutes) (Gerino-Teixeira et al., 2017).

Many factors, including environmental conditions at harvest, precipitation, soil fertility, and cultural practices could influence cooking time of cassava roots and lead to changes in the root’s chemical composition. Roots from the same plant, plants of the same variety, and different genotypes can all have different effects (Oliveira and Moraes, 2009; Reis et al., 2021; Silveira et al., 2021; Santos et al., 2022). Other factors such as the procedures and utensils used for root cooking could also affect the cooking time of clones. Talma et al. (2013) reported average cooking times of 19 and 26 minutes for the BRS Gema de Ovo and Eucalipto clones, respectively, while in the study by Santos et al. (2022), this average cooking time was 26 and 23 minutes, respectively. According to Silveira et al. (2021), different phosphorus doses (between 120 and 240 kg ha^-1^ de P_2_O_5_) increased starch content and reduced cooking time for sweet cassava roots. Besides these external factors, harvest age could also influences cooking time, with earlier-harvested plants showing better cooking ability (Oliveira and Moraes, 2009).

In our study, we examined how environmental factors influenced the performance of different genotypes across various trials (**Supplementary Table S2**). We focused on a group of 20 genotypes evaluated in 3 to 13 trials, each genotype showing cooking capability in at least one trial. In most trials, these genotypes consistently cooked within an average time of 30 minutes or less. However, there were instances where certain genotypes did not cook within the expected time frame in at least one trial. Typically, the harvest and evaluation period span approximately three months (June to August), encompassing the winter and spring seasons when average precipitation tends to be higher. The trials were conducted across four different cities with varying altitudes. **Supplementary Table S2** illustrates that trials where genotypes did not cook as expected were predominantly located in Laje-BA and Sátiro Dias-BA. These locations experienced environmental conditions during the harvest period that did not facilitate the cooking of roots within 40 minutes.

### 5.2 Efficiency of NIR spectroscopy for cooking time classification

The application of NIR spectroscopy to address food classification and plant species has been reported in the literature (Cozzolino et al., 2012; Jadhav et al., 2019; Sousa et al., 2023). In cassava, spectral data collected from root samples, processed dry leaves, and seeds have been used to develop models with high accuracy for the indirect and early classification of genotypes based on starch type (waxy and non-waxy) (Carmo et al., 2019; Sousa et al., 2023). In this study, the classification accuracy for cassava cooking time for binary variables was notably high ( $R_{Cal}^{2}$ ranging from 0.72 to 0.99), showcasing the potential of NIR spectroscopy for classifying cassava clones based on their cooking time. A number of studies have been carried out to predict cooking time in various food crops (Nie et al., 2012; Cichy et al., 2015; Thanathornvarakul et al., 2016; Wafula et al., 2020, 2021). Predictive models based on NIR spectroscopy were developed to forecast cooking time in rice (calibration R^2^≥ 0.81 and validation R^2^≥ 0.87) (Thanathornvarakul et al., 2016). With an R^2^ value of 0.73, the prediction accuracy for cooking time in bean was deemed moderate (Wafula et al., 2021). To the best of our knowledge, this study stands as a pioneering attempt to classify cassava genotypes based on their root cooking time using NIRS spectroscopy. A noteworthy challenge in our dataset lies in its significant class imbalance, where the number of samples varies considerably across different classes. This imbalance can profoundly influence the classification outcomes. To mitigate this issue, we applied the random oversampling technique to the class with the smaller number of samples (those that cook) to achieve a balanced distribution between classes (Johnson and Khoshgoftaar, 2019). This technique is commonly employed in classification problems (Haixiang et al., 2017).

The random oversampling technique has proven effective in addressing imbalances in classification problems involving machine learning and deep learning algorithms, especially in datasets with genotoxicity information and class imbalance (Bae et al., 2021). Random oversampling is comparable to other methods such as synthetic minority oversampling technique (SMOTE), random undersampling, and sample weighting (Chawla et al., 2002; Bae et al., 2021). Hence, the thoughtful combination of machine learning algorithms and data balancing methods is crucial for developing accurate classification models.

The PLS and KNN algorithms have been widely and successfully used for classification analysis (Wafula et al., 2020, 2021; Ye et al., 2022; Meghar et al., 2023; Sousa et al., 2023). In this study, the KNN model demonstrated the most robust classification performance, with accuracies ranging from 0.72 to 0.99 for binary variables and 0.86 to 0.99 for multiclass variables. The extensive dataset under evaluation, combined with the discriminative ability between classes using Euclidean distance, might have contributed to the efficiency of the KNN algorithm. Literature provides ample evidence of KNN effectiveness for multiclass classification (Jadhav et al., 2019). The KNN model showed an accuracy of 83.6% in differentiating between rust, brown spot, frog eye leaf spot, and healthy samples in a study classifying four foliar diseases in soybeans (Jadhav et al., 2019).

The possibility of using NIRS to determine the cooking quality of cassavas roots has been studied (Meghar et al., 2023; Namakula et al., 2023). Namakula et al. (2023) assessed root softness using a penetrometer, a trait significantly influencing cooking time. However, predictions via NIRS were rated as low to moderate, indicating that softness, a force-measured physical parameter, did not correlate well with NIRS spectra (Namakula et al., 2023). A recent study evaluated the potential of near-infrared hyperspectral imaging, which combines NIRS spectroscopy with digital images, in a panel of 31 cassava genotypes to predict cooking quality parameters, dry matter content, water absorption, and texture (Meghar et al., 2023). However, hyperspectral imaging was effective only in predicting dry matter content, indicating a need for protocol improvements and a larger dataset for root texture and water absorption characteristics. In the current study, spectral data were collected from processed fresh root samples, ensuring sample homogenization and avoiding potential spatial distribution variations of organic compounds in the samples of interest. This enabled efficient, high-throughput phenotyping via NIRS for measuring one of the most critical traits in improving sweet cassava.

### 5.3 Binary vs. multiclass classification for cooking time

The classification of genotypes based on cooking time was carried out by considering the variables as both binary and multiclass. Binarization techniques are often developed to address multiclass situations by breaking down the original problem into a binary classification system, simplifying its resolution (Galar et al., 2011). In addition to analyzing the cooking capacity for all (CTC6) or part (CT4C) of the cooking times, we divided the original dataset into subsets of two classes (cooked and uncooked samples) and we calibrate a binary model for each cooking time.

One challenge in multiclass classification is that some algorithms such as logistic regression or support vector machines are only intended for binary classification. To overcome this issue, the KNN and PLS models implemented in the R package *Caret* (Kuhn et al., 2008), which employ the “one-vs-all” strategy was used. In this strategy, a binary classifier is trained for each class, where the positive class is the target class, and the negative class comprises the rest of the classes. In other words, the multiclass variable is treated as a binary variable (Allwein et al., 2000). However, this strategy introduces a higher imbalance between the class being evaluated and the “rest” class, which includes all samples from the other classes.

The findings of this study underscore the benefits and effectiveness of using binary variables to classify cassava genotypes based on cooking time. When considering accuracy between cooking classes, even with the same model and NIR equipment, multiclass variables showed lower variation (50% to 71% for CT4C and 57% to 73% for CT6C) compared to binary variables. In terms of the accuracy of samples that did not cook, the values were 70% and 73%, respectively, for CT4C and CT6C. Despite the increased number of variables, requiring the evaluation of each binary variable individually, binary classification remains more efficient, offering higher classification accuracy for samples and enabling the selection of the most relevant characteristics or variables, such as cooking time ≤ 30 minutes.

The accuracy of the optimal scenario for classifying samples with a cooking time of 30 minutes reached. and $R_{Val}^{2}=0.84$ , with a Kappa value of 0.53. Consequently, it is advised to segregate samples using the NIRS-based classification model for cooking capacity within 30 minutes. This approach enables the assessment of a larger pool of genotypes concerning attributes associated with root quality. Consequently, it facilitates the identification of new sweet cassava varieties possessing pivotal agronomic traits, including high fresh root yield, compatibility with mechanized or semi-mechanized cultivation methods, and resilience against pests and diseases prevalent in the crop.

## 5 Conclusions

This study has demonstrated the potential of using NIRS in conjunction with machine learning techniques to phenotype and classify cassava genotypes based on cooking time. Overall, accuracies ranging from 86% to 99% were achieved for cooking times not greater than thirty minutes. The optimal conditions for developing accurate classification models were given by the K-nearest neighbors (KNN) algorithm analysis of the spectra from the benchtop NIRS equipment NIRFlex, especially when associated with the classification of cooking capacity in binary variables. These findings underscore the potential of NIRS-based phenotyping in enhancing the efficiency of cassava breeding programs, offering a rapid and reliable method for assessing an essential quality trait like cooking time. NIRS phenotyping could be a cost-effective, less labor-intensive, and high-throughput option, that could not only enable processing hundreds of samples per day, but also help reduce phenotyping errors that are commonly associated with conventional methodologies, especially in scenarios where a well-trained team may be lacking, as observed in the “fork test”.

## Data availability statement

The original contributions presented in the study are included in the article/**Supplementary Material**. Further inquiries can be directed to the corresponding author.

## Author contributions

MB: Conceptualization, Data curation, Formal Analysis, Writing – original draft. CM: Data curation, Methodology, Writing – review & editing. EM: Writing – review & editing. CE: Funding acquisition, Project administration, Writing – review & editing. EO: Conceptualization, Funding acquisition, Project administration, Supervision, Writing – review & editing.
