# Supplementary material for: Near infrared spectroscopy for cooking time classification of cassava genotypes
Source: Front Plant Sci. 2024 Jul 12;15:1411772. doi: 10.3389/fpls.2024.1411772 (PMC11272462; doi:10.3389/fpls.2024.1411772)
Supplement: Supplementary file 1 [file DataSheet_1.docx]

**Near infrared spectroscopy (NIR) for cooking time classification of cassava genotypes**

**
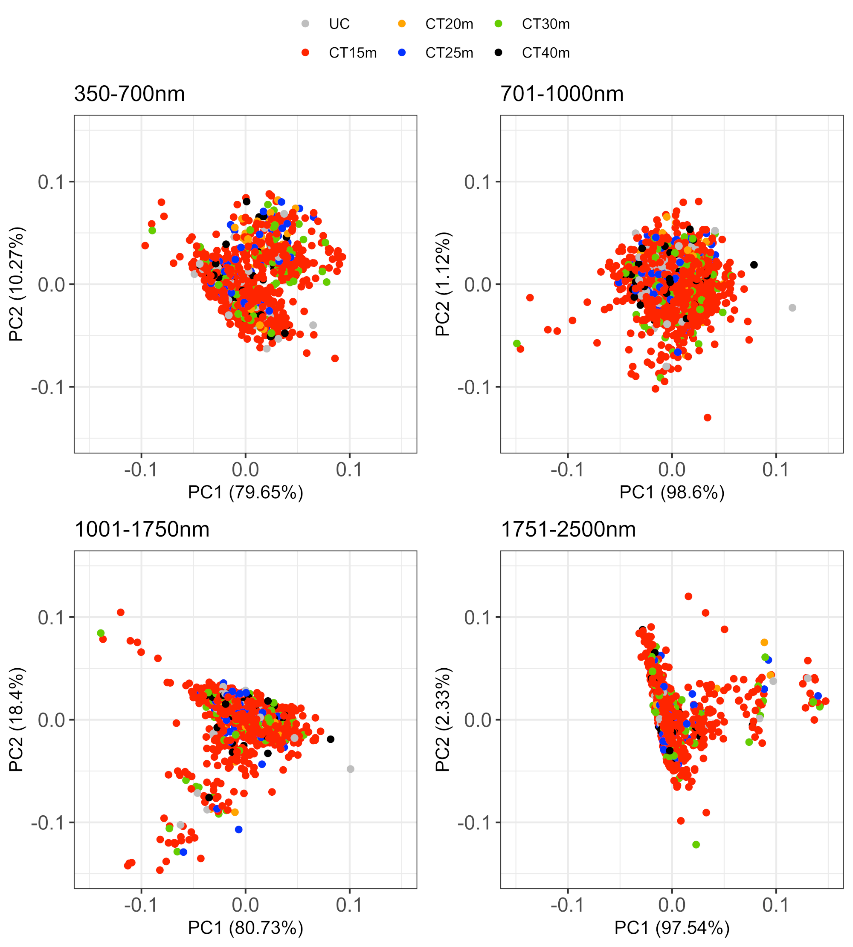
**

**Figure S1**. Principal component analysis of spectral data grouped into four ranges (350-700, 701-1000, 1001-1750, and 1751-2500 nm) collected from fresh roots of 888 cassava accessions belonging to the breeding program of Embrapa Mandioca e Fruticultura using the portable spectrometer QualitySpec® Trek (NIRS QST).


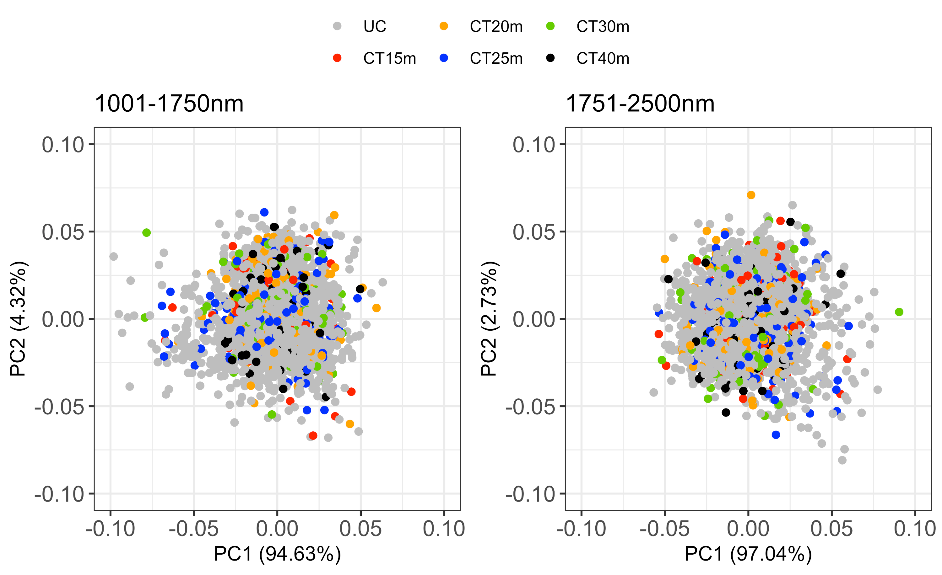


**Figure S2**. Principal component analysis of spectral data grouped into two ranges (1001-1750 and 1751-2500 nm) collected from fresh roots of 888 cassava accessions belonging to the breeding program of Embrapa Mandioca e Fruticultura using the benchtop spectrometer NIRFlex N-500 (NIRFlex).

**
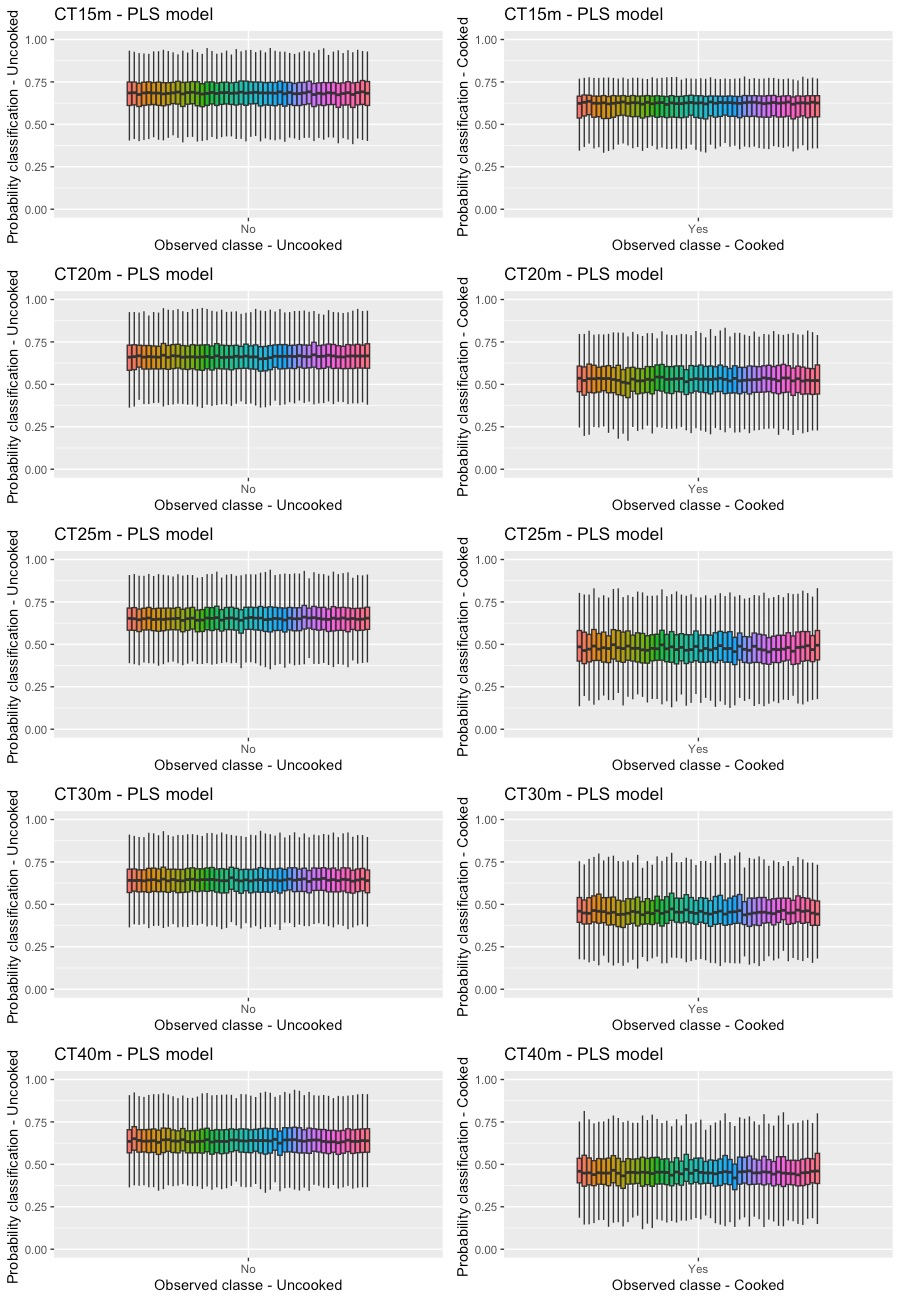
**

**Figura S3.** Boxplot illustrating classification probabilities for each binary variable of cooking time (CT15m, CT20m, CT25m, CT30m, and CT40m) across folds and replications (50 repetitions from cross-validation). The data was analyzed using a PLS (Partial Least Squares) model with near-infrared spectroscopy (NIR) spectra obtained from the QualitySpec® Trek (QST) instrument.


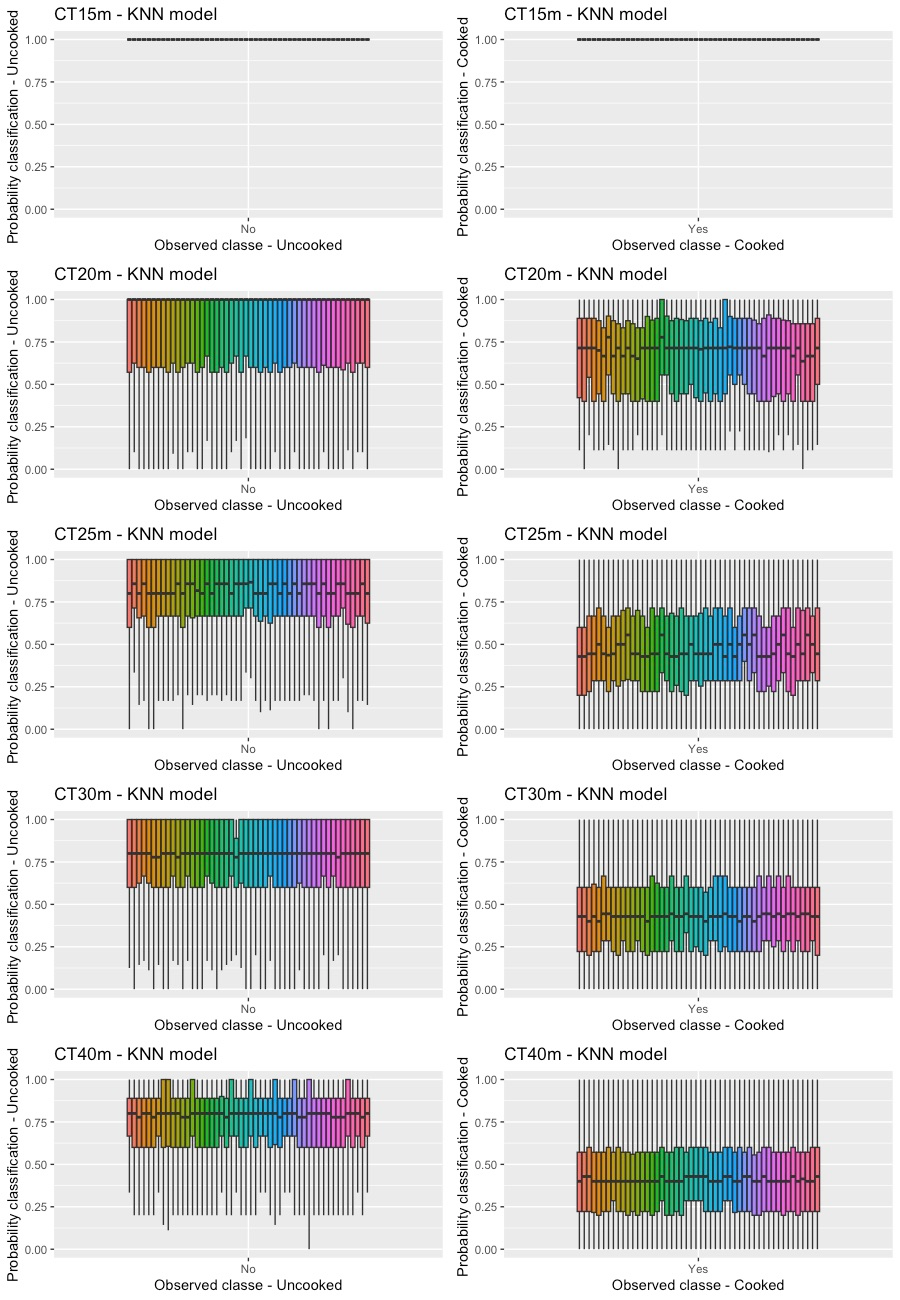


**Figura S4.** Boxplot illustrating classification probabilities for each binary variable of cooking time (CT15m, CT20m, CT25m, CT30m, and CT40m) across folds and replications (50 repetitions from cross-validation). The data was analyzed using a KNN (k-nearest neighbor algorithm) model with near-infrared spectroscopy (NIR) spectra obtained from the QualitySpec® Trek (QST) instrument.


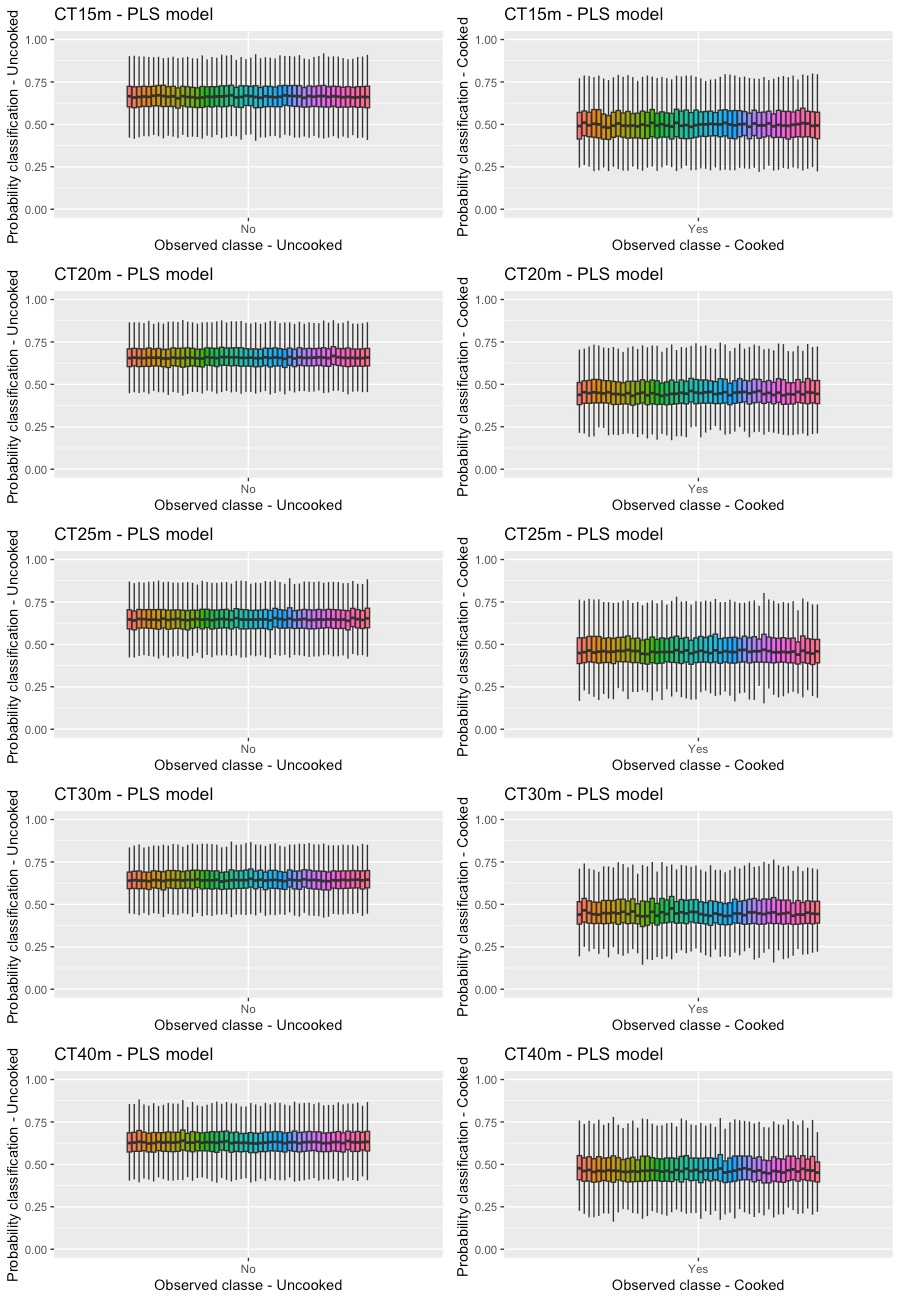


**Figura S5.** Boxplot illustrating classification probabilities for each binary variable of cooking time (CT15m, CT20m, CT25m, CT30m, and CT40m) across folds and replications (50 repetitions from cross-validation). The data was analyzed using a PLS (Partial Least Squares) model with near-infrared spectroscopy (NIR) spectra obtained from the NIRFlex N-500 (NIRFlex) instrument.


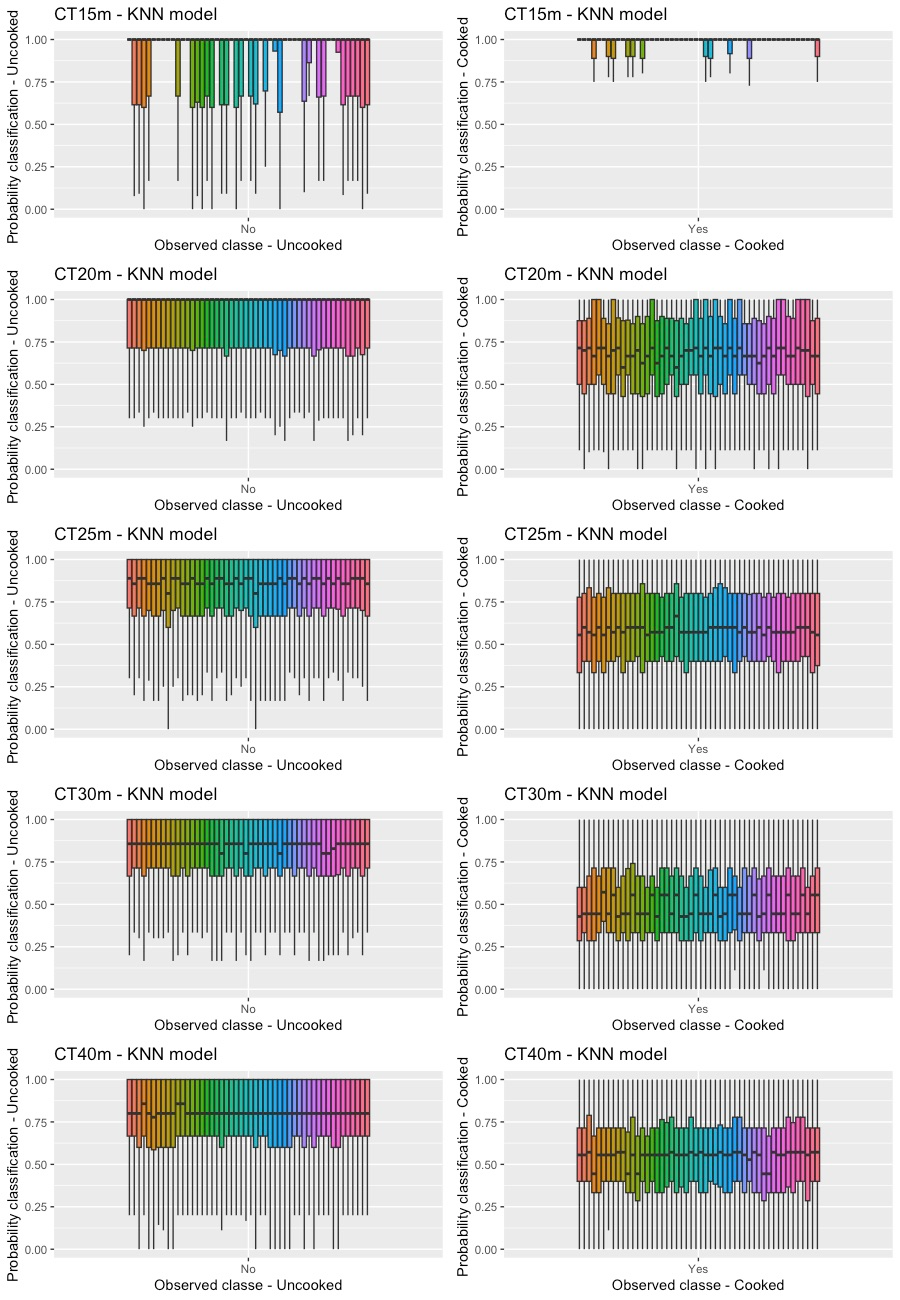


**Figura S6.** Boxplot illustrating classification probabilities for each binary variable of cooking time (CT15m, CT20m, CT25m, CT30m, and CT40m) across folds and replications (50 repetitions from cross-validation). The data was analyzed using a KNN (k-nearest neighbor algorithm) model with near-infrared spectroscopy (NIR) spectra obtained from the NIRFlex N-500 (NIRFlex) instrument.


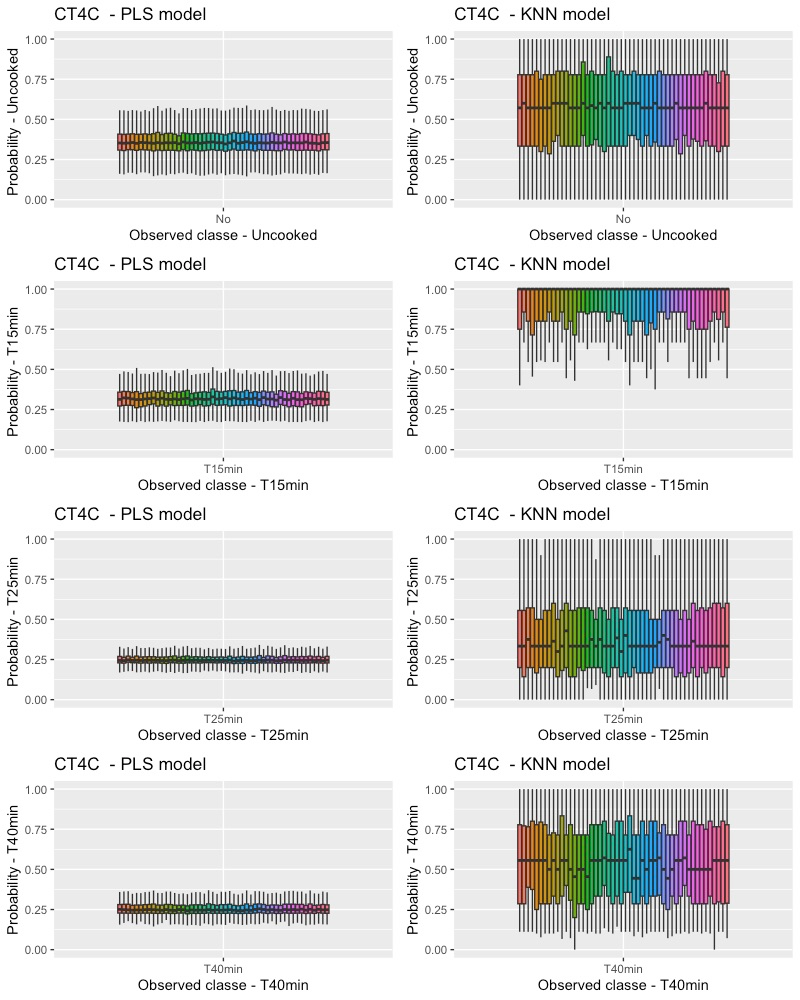


**Figura S7.** Boxplot of classification probabilities for a multiclass cooking time variable with four classes (CT4C) across different folds versus replications (50 repetitions of cross-validation) using Partial Least Squares (PLS) and k-Nearest Neighbor (KNN) models. The models are based on near-infrared spectroscopy (NIR) spectra collected with the QualitySpec® Trek (QST) instrument.


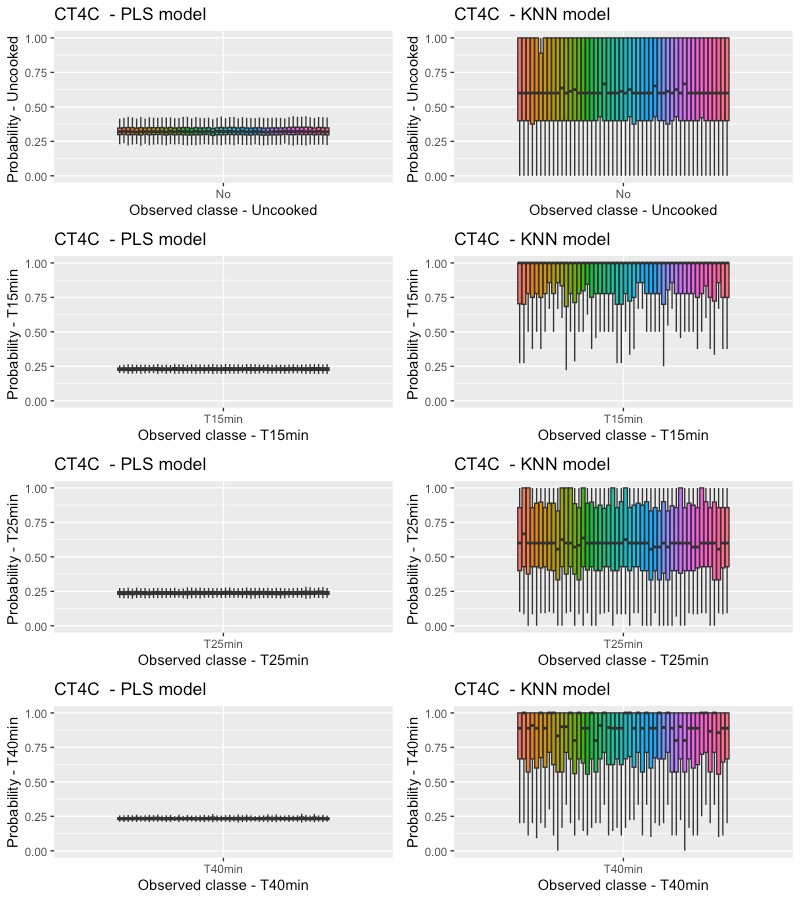


**Figura S8.** Boxplot of classification probabilities for a multiclass cooking time variable with four classes (CT4C) across different folds versus replications (50 repetitions of cross-validation) using Partial Least Squares (PLS) and k-Nearest Neighbor (KNN) models. The models are based on near-infrared spectroscopy (NIR) spectra collected with the NIRFlex N-500 (NIRFlex) instrument.


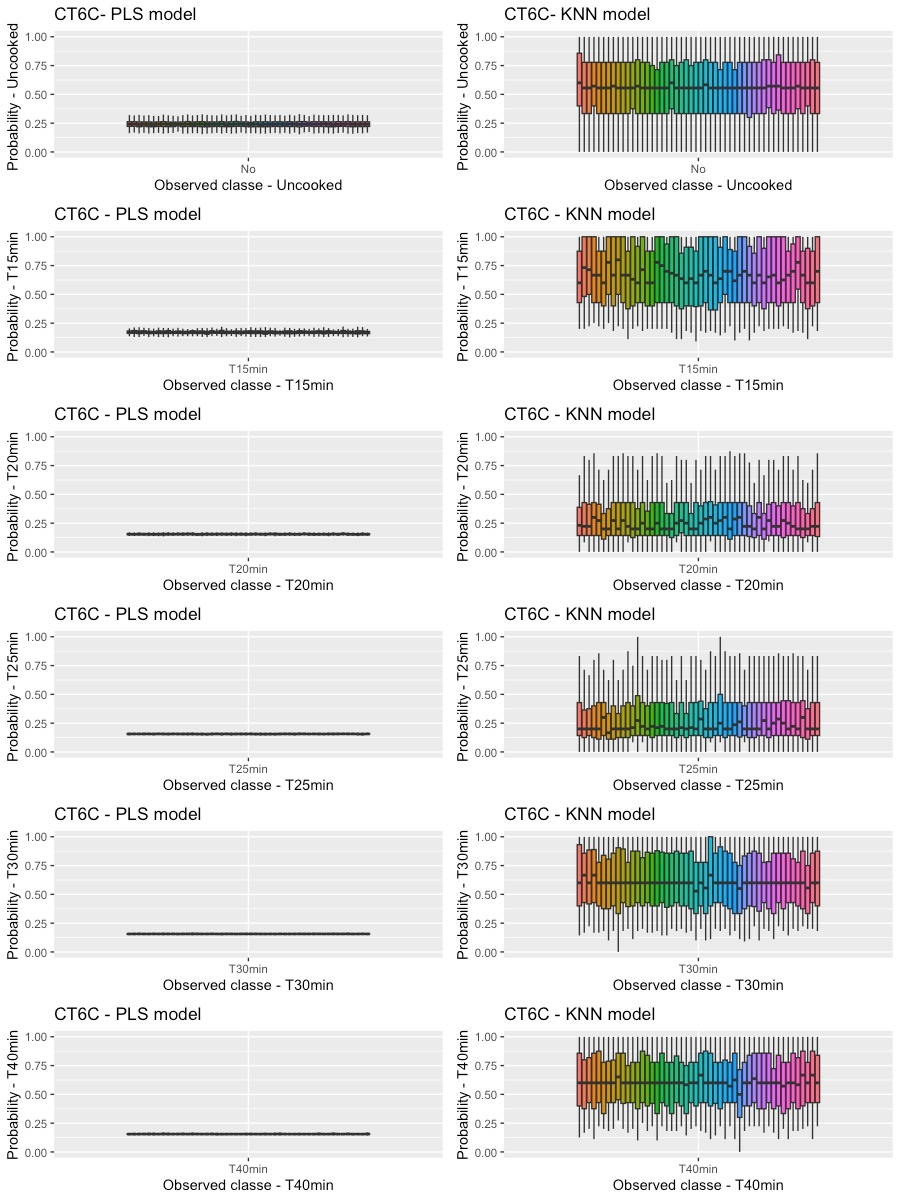


**Figura S9.** Boxplot of classification probabilities for a multiclass cooking time variable with six classes (CT6C) across different folds versus replications (50 repetitions of cross-validation) using Partial Least Squares (PLS) and k-Nearest Neighbor (KNN) models. The models are based on near-infrared spectroscopy (NIR) spectra collected with the QualitySpec® Trek (QST) instrument.


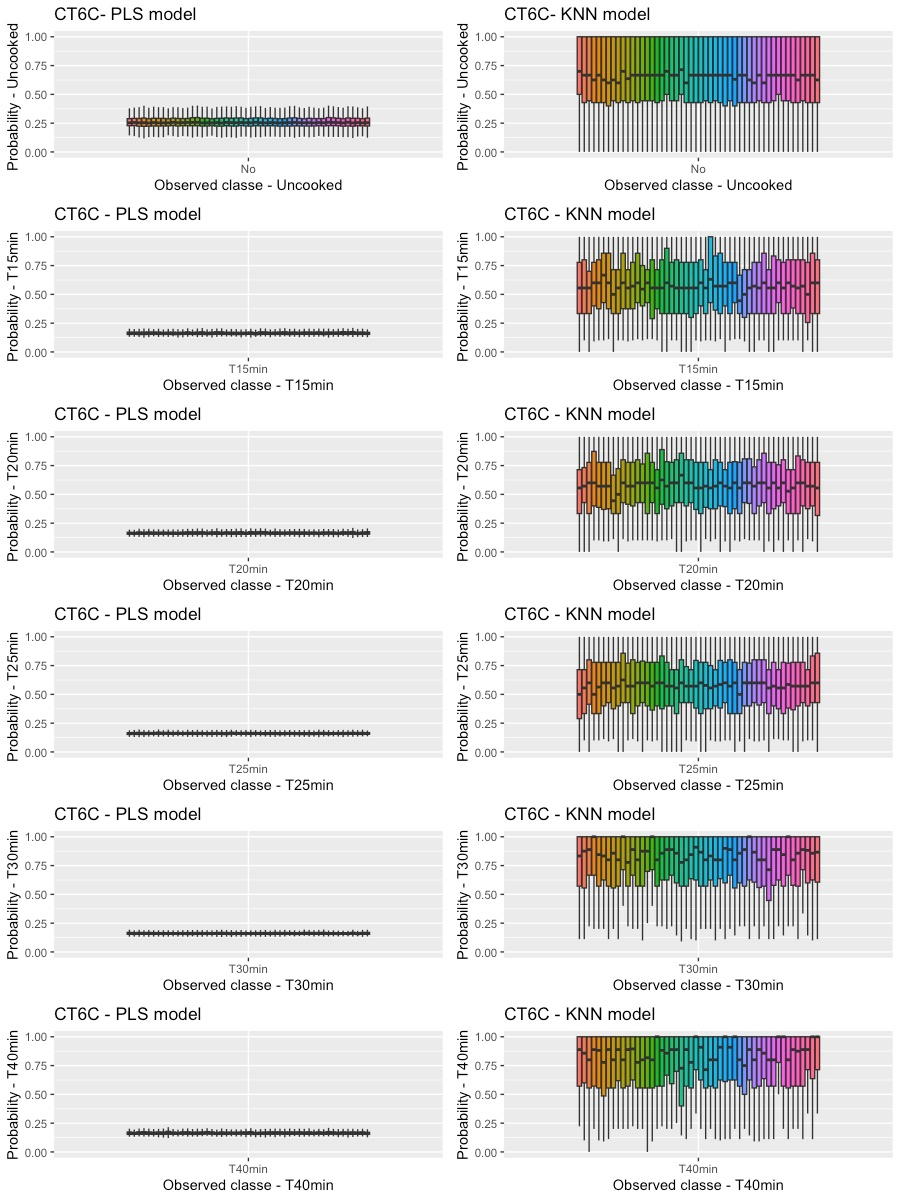


**Figura S10.** Boxplot of classification probabilities for a multiclass cooking time variable with six classes (CT6C) across different folds versus replications (50 repetitions of cross-validation) using Partial Least Squares (PLS) and k-Nearest Neighbor (KNN) models. The models are based on near-infrared spectroscopy (NIR) spectra collected with the NIRFlex N-500 (NIRFlex) instrument.
