# Supplementary material for: Near infrared spectroscopy for cooking time classification of cassava genotypes
Source: Front Plant Sci. 2024 Jul 12;15:1411772. doi: 10.3389/fpls.2024.1411772 (PMC11272462; doi:10.3389/fpls.2024.1411772)
Supplement: Supplementary file 2 [file Table_1.docx]

**Near infrared spectroscopy (NIR) for cooking time classification of cassava genotypes**

**Supplementary Material**

**Table S1**. Mean broad-sense heritability ($H^{2}$) for each wavelength of the NIRS spectra from cassava roots collected in the 17 trials using the NIRFlex N-500 benchtop spectrometer (NIRFlex) and the portable device QualitySpec® Trek (NIRS QST).

| **Trial** | **QST** | |  | **NIRFlex** |
| --- | --- | --- | --- | --- |
|  | $\boldsymbol{H}^{\boldsymbol{2}}$ **(350-2500)** | $\boldsymbol{H}^{\boldsymbol{2}}$ **(1000-2500)** |  | $\boldsymbol{H}^{\boldsymbol{2}}$ **(1000-2500)** |
| BR.AYTGS.20.NH1 | 0.34 | 0.36 |  | 0.44 |
| BR.AYTGS.20.PP1 | 0.18 | 0.21 |  | 0.32 |
| BR.AYTGS.20.RA1 | 0.28 | 0.26 |  | 0.50 |
| BR.CET.20.CNPMF | 0.59 | 0.60 |  | 0.78 |
| BR.CET.20.PP1 | 0.64 | 0.72 |  | 0.61 |
| BR.CETBAG.19.UFRB | - | - |  | 0.73 |
| BR.PTBAG.21.Candeal | 0.65 | 0.71 |  | 0.68 |
| BR.PYT.21.PP1 | 0.70 | 0.72 |  | 0.84 |
| BR.PYT.21.SJ | 0.56 | 0.57 |  | 0.81 |
| BR.PYTGS-C2.21.Emb | 0.55 | 0.64 |  | 0.69 |
| BR.PYTGS-C2.21.NH1 | 0.73 | 0.74 |  | 0.78 |
| BR.PYTGS.19NH2 | 0.35 | 0.37 |  | 0.59 |
| BR.PYTGS.19PP1 | 0.37 | 0.37 |  | 0.42 |
| BR.UYT.21.NH | - | - |  | 0.58 |
| BR.PYTGS.19.RA1 | 0.33 | 0.31 |  | - |
| BR.UYTGS.21.AlaBoaUniao | 0.22 | 0.22 |  | 0.49 |
| BR.UYTGS.21.SDAP | 0.31 | 0.27 |  | 0.56 |

**Table S2**. Average cooking time of some cassava genotypes per trial.

| Genotype | Trial | Mean CT |  |  | Genotype | Trial | Mean CT |  |
| --- | --- | --- | --- | --- | --- | --- | --- | --- |
| BGM-1487 | BR.CETBAG.19.UFRB | 12.5 | < 30 |  | BR-18GS-113.4 | BR.AYTGS.20.NH1 | 0 | UC |
|  | BR.PYTGS.19NH2 | 0 | UC |  |  | BR.AYTGS.20.PP1 | 16.7 | < 30 |
|  | BR.PYTGS.19PP1 | 12.5 | < 30 |  |  | BR.AYTGS.20.RA1 | 0 | UC |
|  | BR.PYTGS.19RA1 | 15.0 | < 30 |  |  | BR.PYTGS.19NH2 | 0 | UC |
| BR-18GS-013.48 | BR.AYTGS.20.NH1 | 30.0 | 30 |  |  | BR.PYTGS.19PP1 | 32.5 | > 30 |
|  | BR.AYTGS.20.PP1 | 6.7 | < 30 |  |  | BR.PYTGS.19RA1 | 20.0 | < 30 |
|  | BR.AYTGS.20.RA1 | 16.7 | < 30 |  | BR-18GS-131.14 | BR.AYTGS.20.PP1 | 15.0 | < 30 |
|  | BR.PYTGS.19NH2 | 0 | UC |  |  | BR.AYTGS.20.RA1 | 0 | UC |
|  | BR.PYTGS.19PP1 | 13.3 | < 30 |  |  | BR.PYTGS.19NH2 | 0 | UC |
|  | BR.PYTGS.19RA1 | 0 | UC |  |  | BR.PYTGS.19PP1 | 15.0 | < 30 |
| BR-18GS-042.27 | BR.AYTGS.20.NH1 | 12.5 | < 30 |  |  | BR.PYTGS.19RA1 | 25.0 | < 30 |
|  | BR.AYTGS.20.PP1 | 21.7 | < 30 |  | BR-18GS-134.11 | BR.AYTGS.20.PP1 | 20.0 | < 30 |
|  | BR.AYTGS.20.RA1 | 13.3 | < 30 |  |  | BR.AYTGS.20.RA1 | 13.3 | < 30 |
|  | BR.PYTGS.19NH2 | 15.0 | < 30 |  |  | BR.PYTGS.19NH2 | 0 | UC |
|  | BR.PYTGS.19PP1 | 18.3 | < 30 |  |  | BR.PYTGS.19PP1 | 8.3 | < 30 |
|  | BR.PYTGS.19RA1 | 20.0 | < 30 |  |  | BR.PYTGS.19RA1 | 20.0 | < 30 |
| BR-18GS-054.17 | BR.AYTGS.20.NH1 | 25.0 | < 30 |  | BRS-Dourada | BR.AYTGS.20.NH1 | 10.0 | < 30 |
|  | BR.AYTGS.20.PP1 | 18.3 | < 30 |  |  | BR.AYTGS.20.PP1 | 20.0 | < 30 |
|  | BR.AYTGS.20.RA1 | 0 | UC |  |  | BR.AYTGS.20.RA1 | 36.7 | > 30 |
|  | BR.PYTGS.19NH2 | 15.0 | < 30 |  |  | BR.CET.20.PP1 | 15.0 | < 30 |
|  | BR.PYTGS.19PP1 | 31.7 | > 30 |  |  | BR.CETBAG.19.UFRB | 5.0 | < 30 |
|  | BR.PYTGS.19RA1 | 30.0 | 30 |  |  | BR.PTBAG.21.Candeal | 8.2 | < 30 |
| BR-18GS-056.12 | BR.AYTGS.20.NH1 | 15.0 | < 30 |  |  | BR.PYT.21.SJ | 0 | UC |
|  | BR.AYTGS.20.PP1 | 25.0 | < 30 |  |  | BR.PYTGS.19NH2 | 0 | UC |
|  | BR.AYTGS.20.RA1 | 0 | UC |  |  | BR.PYTGS.19PP1 | 22.5 | < 30 |
|  | BR.PYTGS.19NH2 | 0 | UC |  |  | BR.PYTGS.19RA1 | 20.0 | < 30 |
|  | BR.PYTGS.19PP1 | 28.3 | < 30 |  |  | BR.UYT.21.NH | 15.0 | < 30 |
|  | BR.PYTGS.19RA1 | 0 | UC |  |  | BR.UYTGS.21.AlaBoaUniao | 0 | UC |
| BR-18GS-056.9 | BR.AYTGS.20.NH1 | 0 | UC |  |  | BR.UYTGS.21.SDAP | 0 | UC |
|  | BR.AYTGS.20.PP1 | 26.7 | < 30 |  | BRS-Gema-de-Ovo | BR.AYTGS.20.PP1 | 22.5 | < 30 |
|  | BR.AYTGS.20.RA1 | 0 | UC |  |  | BR.AYTGS.20.RA1 | 25.0 | < 30 |
|  | BR.PYTGS.19NH2 | 0 | UC |  |  | BR.CET.20.CNPMF | 15.5 | < 30 |
|  | BR.PYTGS.19PP1 | 12.5 | < 30 |  |  | BR.CET.20.PP1 | 20.0 | < 30 |
|  | BR.PYTGS.19RA1 | 30.0 | 30 |  |  | BR.PTBAG.21.Candeal | 1.8 | < 30 |
| BR-18GS-084.22 | BR.AYTGS.20.PP1 | 16.7 | < 30 |  |  | BR.PYT.21.PP1 | 0 | UC |
|  | BR.AYTGS.20.RA1 | 6.7 | < 30 |  |  | BR.PYT.21.SJ | 0 | UC |
|  | BR.PYTGS.19NH2 | 0 | UC |  |  | BR.PYTGS-C2.21.Emb | 25.0 | < 30 |
|  | BR.PYTGS.19PP1 | 23.3 | < 30 |  |  | BR.PYTGS.19NH2 | 0 | UC |
|  | BR.PYTGS.19RA1 | 25.0 | < 30 |  |  | BR.PYTGS.19PP1 | 35.0 | > 30 |
| BR-18GS-085.83 | BR.AYTGS.20.NH1 | 10.0 | < 30 |  |  | BR.UYT.21.NH | 20.0 | < 30 |
|  | BR.AYTGS.20.PP1 | 26.7 | < 30 |  |  | BR.UYTGS.21.AlaBoaUniao | 0 | UC |
|  | BR.AYTGS.20.RA1 | 0 | UC |  |  | BR.UYTGS.21.SDAP | 0 | UC |
|  | BR.PYTGS.19NH2 | 0 | UC |  | BRS-Jari | BR.AYTGS.20.RA1 | 8.3 | < 30 |
|  | BR.PYTGS.19PP1 | 21.7 | < 30 |  |  | BR.CET.20.PP1 | 15.0 | < 30 |
| BR-18GS-111.25 | BR.AYTGS.20.PP1 | 13.3 | < 30 |  |  | BR.PTBAG.21.Candeal | 1.9 | < 30 |
|  | BR.AYTGS.20.RA1 | 0 | UC |  |  | BR.PYTGS.19NH2 | 0 | UC |
|  | BR.PYTGS.19NH2 | 0 | UC |  |  | BR.PYTGS.19PP1 | 11.7 | < 30 |
|  | BR.PYTGS.19PP1 | 6.7 | < 30 |  |  | BR.PYTGS.19RA1 | 40.0 | > 30 |
|  | BR.PYTGS.19RA1 | 20.0 | < 30 |  |  | BR.UYT.21.NH | 0 | UC |
| BR-18GS-111.45 | BR.AYTGS.20.NH1 | 30.0 | 30 |  |  | BR.UYTGS.21.AlaBoaUniao | 8.3 | < 30 |
|  | BR.AYTGS.20.PP1 | 10.0 | < 30 |  |  | BR.UYTGS.21.SDAP | 0 | UC |
|  | BR.AYTGS.20.RA1 | 0 | UC |  | Eucalipto | BR.CET.20.PP1 | 15.0 | < 30 |
|  | BR.PYTGS.19NH2 | 0 | UC |  |  | BR.PYT.21.SJ | 20.0 | < 30 |
|  | BR.PYTGS.19PP1 | 18.3 | < 30 |  |  | BR.PYTGS.19PP1 | 17.5 | < 30 |
|  | BR.PYTGS.19RA1 | 30.0 | 30 |  |  | BR.UYT.21.NH | 17.5 | < 30 |
| BR-18GS-111.78 | BR.AYTGS.20.NH1 | 0 | UC |  |  | BR.UYTGS.21.SDAP | 0 | UC |
|  | BR.AYTGS.20.PP1 | 6.7 | < 30 |  | BR-18GS-054.2 | BR.PYTGS.19NH2 | 32.5 | > 30 |
|  | BR.AYTGS.20.RA1 | 0 | UC |  |  | BR.PYTGS.19PP1 | 22.5 | < 30 |
|  | BR.PYTGS.19NH2 | 20.0 | < 30 |  |  | BR.PYTGS.19RA1 | 20.0 | < 30 |
|  | BR.PYTGS.19PP1 | 5.0 | < 30 |  |  |  |  |  |
